# Impact of alcohol consumption on tuberculosis treatment outcomes: a prospective longitudinal cohort study protocol

**DOI:** 10.1186/s12879-018-3396-y

**Published:** 2018-09-29

**Authors:** Bronwyn Myers, Tara C Bouton, Elizabeth J Ragan, Laura F White, Helen McIlleron, Danie Theron, Charles D H Parry, C Robert Horsburgh, Robin M Warren, Karen R Jacobson

**Affiliations:** 1 Alcohol, Tobacco and Other Drug Research Unit, South African Medical Research Council, Cape Town, South Africa; 2 Division of Infectious Diseases, Brown University Alpert School of Medicine, Providence, RI USA; 3 Section of Infectious Diseases, Boston University School of Medicine, 801 Massachusetts Avenue, 2nd floor, Crosstown Center, Boston, MA 02118 USA; 4 Department of Biostatistics Boston University School of Public Health, Boston, MA USA; 5 Division of Clinical Pharmacology, Department of Medicine, University of Cape Town, Cape Town, South Africa; 6 Brewelskloof Hospital, Worcester, South Africa; 7 Department of Medicine, Boston University School of Medicine, Boston, MA USA; 8 Departments of Epidemiology, Biostatistics and Global Health, Boston University School of Public Health, Boston, MA USA; 9 Department of Science and Technology, National Research Foundation Centre of Excellence in Biomedical Tuberculosis Research, South Africa Medical Research Council for Molecular Biology and Human Genetics, Stellenbosch University, Tyberberg, South Africa

**Keywords:** Tuberculosis, *Mycobacterium tuberculosis*, Alcohol, Pharmacokinetics, Treatment adherence, Treatment outcome

## Abstract

**Background:**

An estimated 10% of tuberculosis (TB) deaths are attributable to problematic alcohol use globally, however the causal pathways through which problem alcohol use has an impact on TB treatment outcome is not clear. This study aims to improve understanding of these mechanisms. Specifically, we aim to 1) assess whether poor TB treatment outcomes, measured as delayed time-to-culture conversion, are associated with problem alcohol use after controlling for non-adherence to TB pharmacotherapy; and 2) to determine whether pharmacokinetic (PK) changes in those with problem alcohol use are associated with delayed culture conversion, higher treatment failure/relapse rates or with increased toxicity.

**Methods:**

Our longitudinal, repeated measures, prospective cohort study aims to examine the associations between problem alcohol use and TB treatment outcomes and to evaluate the effect of alcohol on the PK and pharmacodynamics (PD) of TB drugs. We will recruit 438 microbiologically confirmed, pulmonary TB patients with evidence of rifampicin susceptibility in Worcester, South Africa with 200 HIV uninfected patients co-enrolled in the PK aim. Participants are followed for the six months of TB treatment and an additional 12 months thereafter, with sputum collected weekly for the first 12 weeks of treatment, alcohol consumption measures repeated monthly in concert with an alcohol biomarker (phosphatidylethanol) measurement at baseline, and in person directly observed therapy (DOT) using real-time mobile phone-based adherence monitoring. The primary outcome is based on time to culture conversion with the second objective to compare PK of first line TB therapy in those with and without problem alcohol use.

**Discussion:**

Globally, an urgent need exists to identify modifiable drivers of poor TB treatment outcomes. There is a critical need for more effective TB treatment strategies for patients with a history of problem alcohol use. However, it is not known whether poor treatment outcomes in alcohol using patients are solely attributable to noncompliance. This study will attempt to answer this question and provide guidance for future TB intervention trials.

**Trial registration:**

Clinicaltrials.gov Registration Number: NCT02840877. Registered on 19 July 2016.

**Electronic supplementary material:**

The online version of this article (10.1186/s12879-018-3396-y) contains supplementary material, which is available to authorized users.

## Background

Tuberculosis (TB) is the leading cause of death due to an infectious disease globally. In 2016 there were an estimated 10.4 million new TB cases, of which 4.7% were attributable to harmful use of alcohol [1]. Problem alcohol use, a volume or pattern of alcohol use resulting in adverse health outcomes, is a key driver of poor TB treatment response [2]. In comparison to patients who do not consume alcohol, those who do consume alcohol, and especially those who engage in heavy episodic drinking, have been shown to have delayed culture conversion and higher rates of treatment failure, relapse and death [3].

The causal pathways by which problem alcohol use affect TB treatment response, however, are poorly understood. This is due largely to difficulties in studying patients with alcohol-related problems and a lack of detailed data on their TB medication adherence. Heavy alcohol use impacts retention in care and is associated with missed DOT visits [4], with one study showing that MDR TB patients who consumed alcohol during treatment on average missed 18 more intensive phase doses [5]. In order to improve individual TB treatment outcomes, decrease transmission, and guide future treatment intervention strategies, we urgently need an improved understanding of the relationship between alcohol and TB. One potential mechanism for worse TB outcomes is poor treatment adherence and loss to follow up [2], yet observational and animal studies suggest biological mechanisms are also contributing to the association between alcohol use and poor TB clinical outcomes.

Mouse models have shown that compared to controls, ethanol-consuming mice have significantly higher mycobacterial burden and impaired granuloma formation [6], as well as impaired response to BCG vaccination [7]. Additionally, alcohol has been shown to inhibit phagocytic and bactericidal activity of macrophages [8], decrease the number and function of dendritic cells [9] and neutrophils [10], and modulate T cell function [9], B cells, cytokine production and the interferon gamma pathway [6]. In vitro and in vivo data support the impact of alcohol on the immune system as a biological mechanism for poor TB clinical outcomes in those who drink alcohol. Furthermore, chronic heavy drinking is associated with inhibition of phagocytosis and decreased production of growth factors amongst innate immune cells in a dose and time dependent manner [11], suggesting that chronic alcohol use has a greater detrimental effect on the immune response to TB. While these models support the concept that problem alcohol users who consistently take their medications will have worse treatment outcomes than those without problem alcohol use, this hypothesis must still be confirmed in human studies.

Another hypothesized biological mechanism to explain the harmful impact of problem alcohol use on TB clinical outcomes is alcohol’s influence on the pharmacokinetics (PK) and pharmacodynamics (PD) of TB drugs. A recent meta-analysis concluded that studies with lower default rates did not differ significantly in microbiologic failure, acquired drug resistance, or relapse compared to studies with higher default rates, suggesting that rather than adherence, the bioavailability of antituberculosis medications plays an important role in TB outcomes [12]. Furthermore, using the TB hollow-fiber system model in concert with Monte Carlo simulations modelling of degrees of noncompliance, a recent study showed poor adherence was associated with microbiologic failure only when nonadherence exceeded 60%, similarly supporting PK variability as the explanation for acquired drug resistance [13]. While still theoretical for comorbid problem alcohol use, studies of patients with TB and HIV have shown that decreased antitubercular drug absorption [14] and altered PK leads to TB drug resistance [15].

Alcohol has been shown to alter the intestinal absorption of second-line antituberculosis medications [16, 17]; however, the pathway to bioavailability is further complicated by protein binding and first pass metabolism which may also prove to be affected by alcohol use. Studies on the impact of alcohol on the bioavailability of isoniazid have been contradictory [18–20], while a study of patients with slow clinical response to treatment, failure or early relapse found that alcohol use was associated with higher rifampin serum concentrations [21]. A detailed analysis of the PK and PD of all four TB drugs in patients with problem alcohol use will allow for an improved understanding of which TB drugs are most impacted, leading to optimized dosing and treatment duration or possible substitutions for individual drugs that are consistently performing poorly in this population at high risk for resistance and treatment failure. The goal of this study is therefore to clarify the causal mechanisms underlying the deleterious effects of problem alcohol use on TB treatment response.

### Primary objectives

The Tuberculosis Treatment and Alcohol Use Study (TRUST) has two specific aims. The first is to assess whether poor TB treatment outcomes, measured as delayed time-to-culture conversion, are associated with problem alcohol use after controlling for non-adherence. We hypothesize that poor TB treatment outcomes are associated with problem alcohol use and that end organ damage due to chronic alcohol use, rather than effects of acute ingestion, will be the strongest predictor of poor treatment outcomes after controlling for adherence.

The second aim is to compare the PK of anti-tuberculous medications in those with and without problem alcohol use, and to determine whether these PK changes are associated with delayed culture conversion, higher treatment failure/relapse rates, or with increased toxicity. We hypothesize that persons with problem alcohol use will have altered peak drug concentration (Cmax) and area under the curve (AUC) due to alcohol’s effects on drug absorption and metabolism and that these PK changes will be associated with poor clinical outcomes and increased toxicity.

## Methods

**Fig. 1:**
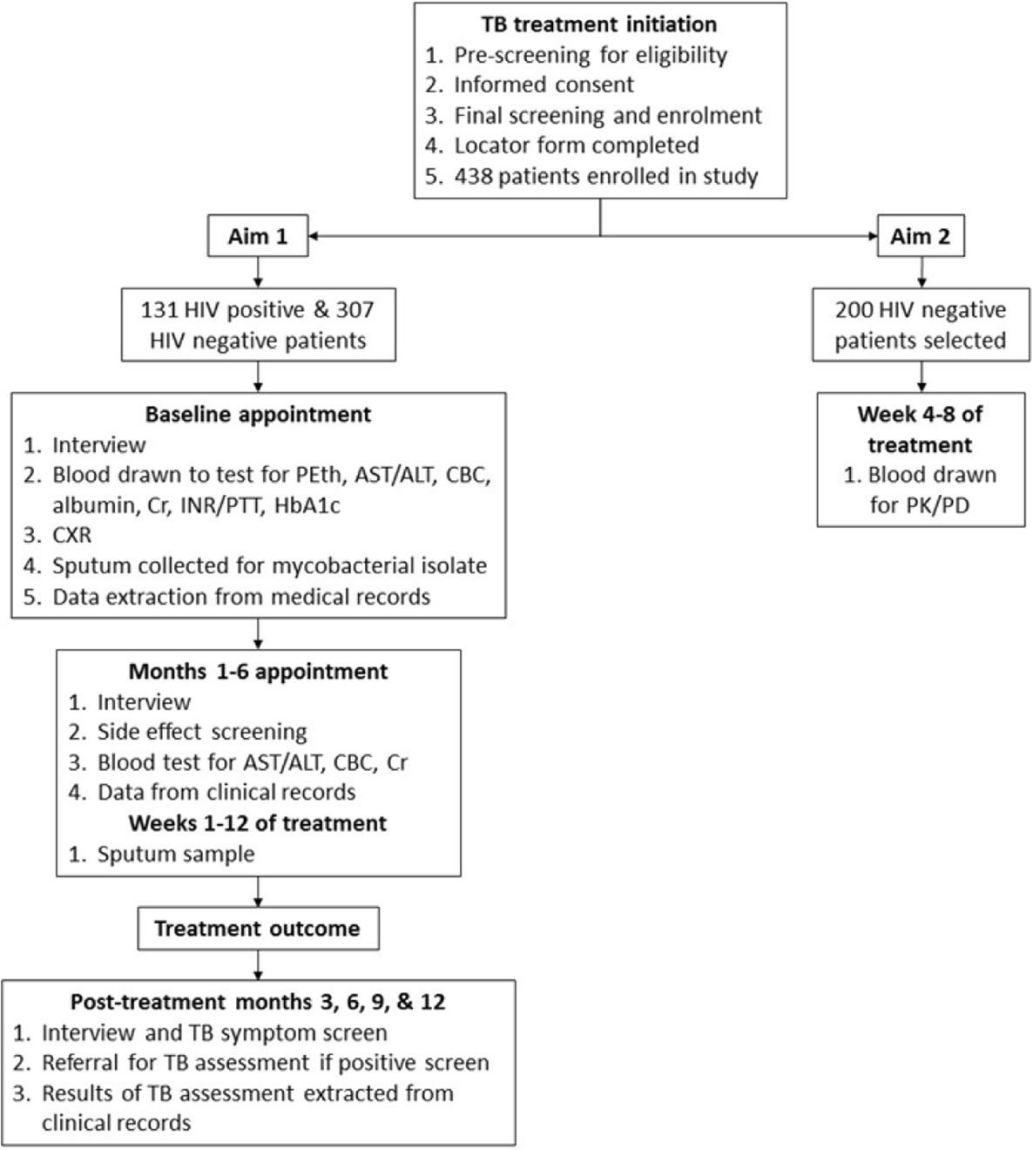
TRUST study schedule for participant follow up

TRUST is a prospective, longitudinal, repeated-measures study which plans to recruit 438 culture-positive, pulmonary TB participants in Worcester, South Africa and follow them over an 18-month period (6-month treatment period, 12 months post-treatment; Fig. 1).

### Study site selection

This study is recruiting participants receiving treatment for TB from the Worcester Community Day Centre, a clinic in Worcester which is the main town of the Breede Valley municipality in the Western Cape Province of South Africa. In this province, TB is the leading cause of natural death [22] and, in South Africa in general, the TB epidemic is intertwined with problem alcohol use. Between 2000 and 2014, alcohol-attributable TB incidence increased by more than 100%, a greater increase than any other country studied and more than five times higher than the global average [23]. In the semirural farming community of Worcester, problem alcohol use is common [24]. A recent study reported an incidence of 16–19% in females and 33–56% in males in this area [25]. The legacy of the “dop” system, whereby farm workers were paid in part with wine, has led to a pattern of heavy, episodic drinking in this farming community [26]. The high prevalence of alcohol problems and TB makes this health district a uniquely appropriate setting to study how alcohol use impacts TB outcomes.

### Participant eligibility criteria

Individuals initiating treatment for smear, Xpert or culture positive drug susceptible pulmonary TB, who are willing to participate and provide written informed consent, are at least 15 years old and expected to remain in the local area for the next 2 years are eligible to participate in this study. Participants will be excluded from this study if they have rifampin resistance, epilepsy, have a contraindication to start on standard 4-drug TB therapy, have been treated for TB in the last two years, have unknown HIV status and are unwilling to be tested, or if they are pregnant at study enrollment (Table 1). In addition, participants who are HIV positive are excluded from the pharmacokinetic cohort (Aim 2 only).

**Table 1:** TRUST Participant Inclusion and Exclusion Criteria

Inclusion Criteria	Exclusion Criteria
● At least 15 years old	● Treated for TB in the last 2 years (defined as at least 1 month of TB treatment)
● Initiating TB treatment	● Have RIF-resistant TB (RIF resistance will be known at screening from Xpert MTB/RIF)
● Expect to remain in the local area for the next 2 years	● Extrapulmonary TB
● Agree to comply with all study requirements, including provision of contact information and attendance at all study appointments	● Contraindication to start on standard 4-drug therapy
● Provide written, informed consent to participate in the study if ≥18 years of age or written individual consent and separate parental consent if < 18 years	● No documented HIV status from the previous 6 months and refuse HIV testing
	● Pregnant at study enrollment
	● History of epilepsy
	● Do not speak English or Afrikaans
	● HIV seropositive (for Aim 2 only)

Abbreviations: *TB*, tuberculosis, *RIF*, rifampin

### Recruitment and assessment of primary objectives

Study fieldworkers will approach all patients initiating TB treatment who meet the study’s eligibility criteria for screening and potential enrollment. If the patient is eligible and willing to participate, then written informed consent is obtained on the same day as TB treatment initiation. Immediately following study enrollment, a study nurse collects socio-demographic information and conducts behavioral assessments of the participant including participant questionnaires on lifetime and recent alcohol use, which is confirmed through biomarker analysis, further described below. Participants identified as having problem alcohol use are offered a referral to local substance abuse treatment services. If not completed by the clinic as part of the work-up for the current episode of TB disease, all participants receive chest radiographs. A baseline sputum specimen is collected for acid fast bacilli (AFB) smear and mycobacterial culture along with minimal inhibitory concentrations (MICs) for isoniazid, rifampin, ethambutol, and pyrazinamide, and blood samples are drawn to assess complete blood count (CBC), alanine transaminase (ALT), aspartate transaminase (AST), creatinine (Cr), prothrombin time (PT)/international normalized ratio (INR), albumin, and hemoglobin A1C (HbA1c) (Table 2). From treatment records of study participants, clinical data on previous TB disease, Xpert MTB/RIF result, AFB smear results, mycobacterial culture result, HIV status, and medical history is extracted. For the primary outcomes for Aim 1 and 2, participants provide weekly first morning sputum samples during the first 12 treatment weeks to assess rate of sterilization/culture conversion. Follow up interviews are performed monthly for the 6 treatment months during which a brief interviewer-administered questionnaire on alcohol use and TB medication side effects evaluation is performed by the study nurse. If there is a positive side effect screen at any appointment, blood is drawn to test for CBC, Cr, ALT and AST. A final sputum specimen is collected after completion of five months of treatment to assess treatment outcome, as defined by the WHO. After treatment completion (as defined by the clinic), participants are interviewed at 3, 6, 9, and 12 months post-treatment regarding alcohol use (Table 2) and screened for any re-occurrence of TB symptoms. Those with a positive TB symptom screen are referred to the clinic for microbiologic assessment.

**Table 2:** SPIRIT Figure

		STUDY PERIOD										
	Enrolment	Allocation	During Treatment Months						Post Treatment Months			
TIMEPOINT	*−* _*1*_	0	*1*	*2*	*3*	*4*	*5*	*6*	*3*	*6*	*9*	*12*
ENROLMENT:												
Eligibility screen	X											
Informed consent	X											
*Chest X-Ray*		X										
*HIV Testing*	X											
*Urine pregnancy test*	X											
Allocation		X										
INTERVENTIONS:												
*Weekday DOT & weekend self-report*		X	X	X	X	X	X	X				
*PK/PD evaluation*^a^				X								
*Sputum specimens*^b^		X	X	X	X		X					
*Blood specimens*^c^		X										
ASSESSMENTS:												
*Side effects screen*			X	X	X	X	X	X				
*TLFB*		X	X	X	X	X	X	X	X	X	X	X
*Behavioral Assessment*^d^		X						X		X		X
*TB Symptom Screen*									X	X	X	X

^a^The PK/PD visit is completed between weeks 4 and 8 of treatment for the Aim 2 cohort only

^b^Collected during screening, then weekly for weeks 1–12 of treatment, with a final specimen at month 5

^c^Blood is drawn at the initial visit for complete blood count (CBC), alanine transaminase (ALT) and aspartate transaminase (AST), albumin, creatinine (Cr), international normalized ratio (INR), and partial thromboplastin time (PTT), hemoglobin A1c (HbA1c) and Phosphatidylethanol (PEth). In the event of a positive side effect screen, the following levels will be reassessed: assessed in the event of a positive side effect screen: Complete blood count (CBC), alanine transaminase (ALT) and aspartate transaminase (AST), and creatinine (Cr)

^d^The behavioral assessment includes Fagerstrom, AUDIT, DUDIT, CES-D and Household Hunger Screening

For Aim 2, 200 HIV-negative participants from the original cohort are co-enrolled in a single 8-h session with intensive PK/PD studies of isoniazid, rifampin, ethambutol, and pyrazinamide during treatment weeks 4–8, that is after the patient has completed one month of treatment and before they transition to the continuation phase of treatment. During this session, the antituberculosis drugs are administered by the study nurse under fasting conditions and blood samples are obtained immediately before the dose, then again at 1.5, 3, 5, and 8 h after the dose is ingested. Plasma samples are stored frozen at − 80 °C freezer until analysis.

### Evaluation of alcohol and other substance use

TRUST uses detailed, repeated measure documentation of participant medication adherence and alcohol consumption during TB treatment to identify alcohol-related biologic causal mechanisms that interfere with TB treatment response. Drinking behavior of participants is assessed in the form of frequency, quantity, volume, patterns and types of alcohol consumption using the Alcohol Timeline Follow Back (TLFB) technique [27]. Additionally, the 10-item Alcohol Use Disorders Identification Test (AUDIT) is used to assess lifetime hazardous and harmful patterns of alcohol use [28]. The Fagerström Test for Nicotine Dependence (FTND) is administered to assess current tobacco use and dependence [29], and in addition to dedicated illicit drug history taking, the 11-item Drug Use Disorders Identification Test (DUDIT) is administered to assess for harmful patterns of illicit drug use and dependence [30]. Depression is assessed with the Center for Epidemiologic Studies Depression Scale (CES-D) [31] and the Household Hunger Scale (HHS) is used to assess food security [32]. The innovative repeated measures design (Table 2) enables us to capture alcohol use changes over the course of treatment, as for example, we hypothesize that patients may report less alcohol use around the time of treatment initiation when they are feeling ill and then return to drinking heavier amounts as their health improves. Additionally, self-report, on the various baseline alcohol measures described above, is validated using phosphatidylethanol (PeTH) measurement at the baseline assessment. PeTH is a biomarker of alcohol consumption in the preceding 30 days and will be measured from dried blood spots sent to United States Drug Testing Laboratories in Des Plaines, IL [33]. By validating standardized, repeated alcohol measures with a baseline blood based biomarker, we aim to substantially reduce misclassification of alcohol use compared with studies which to date have largely relied solely on chart biopsy.

#### Evaluation of adherence

In order to capture comprehensive data on adherence in those with problem alcohol use, TRUST has developed a system for patient engagement including active tracking of participants with daily documentation of their pill-taking by a team of study-employed DOT workers, coupled with frequent reminders and graded subject reimbursements based on adherence. During the 6-month treatment period, pill-taking is documented by these DOTs workers using smartphones on weekdays and self-report is collected on Mondays to document the preceding weekend compliance. DOT workers submit adherence data to a central server on a daily basis for regular review of cumulative indicators by the TRUST team, allowing for real-time identification of participants needing increased outreach to remain engaged in care. DOT workers also collect the weekly sputum specimens during the first 12 weeks of treatment so that the onus is not on the participant to deliver the specimens. In addition to reimbursement for regular study visits, TRUST employs graded reimbursement in the form of grocery vouchers based on percent of DOT visits and weekly sputa captured successfully during the first three months of treatment.

### Analysis

#### Sample size assumptions

In recent studies in Western Cape Province, 20–30% of patients on standard therapy had mycobacterial growth in MGIT sputum cultures after 8 weeks of therapy (personal communication, Mark Hatherill, MD) with roughly 50–60% of TB patients in the region reporting recent alcohol use [34, 35]. Our goal enrollment of 438 patients is based on a logrank test to estimate the power to detect the difference in time to positive sputum culture with and without problem alcohol consumption.

More specifically, with 291 participants (87 participants being problem alcohol drinkers), assuming a 5% drop-out rate at 12 weeks, and varying levels of survival among those without problematic alcohol consumption and 80% power, if 15% of those without problematic alcohol have a positive culture at 12 weeks, then we will be able to detect a HR of 0.63. In order to be able to stratify participants based on treatment adherence (those taking < 80% of medication doses), we inflated the 291 by 5% and then inflated by an additional 30% for HIV positive participants. This would mean recruiting 438 participants for Aim 1 (307 HIV negative). For Aim 2, we used the same model and aim to recruit 200 participants from the 307 HIV negative, omitting any individuals who do not want the full day of phlebotomy or not able to participate due to their schedule. This will allow us to detect a HR of 0.57, indicating 0.34 in the alcohol group having positive cultures at 12 weeks.

#### Assessment of the study endpoints/primary objectives

For the primary analysis, problem alcohol use will be assessed using both continuous and categorical measures to determine the frequency of acute exposure and the regularity of alcohol ingestion. We will use basic bivariate tests to assess the association between alcohol use and the primary TB outcomes (time to positivity at 12 weeks), as well as the association between potential confounders and problem alcohol use. We will investigate the role of mediating factors by estimating the direct, indirect, and total effects of alcohol use with nonlinear models on our outcomes of time to positivity (TTP) and poor TB outcomes, including death, treatment failure, and relapse. We will control for HIV as a potential confounder and, if necessary, stratify our results by HIV status. Using poisson, negative binomial, or Cox proportional hazards models as appropriate, we will model TTP and poor TB outcomes. Additionally, we will investigate potential threshold effects of alcohol by using a penalized spline for alcohol in the model and assessing nonlinear patterns graphically and with statistical tests. We will further investigate the distinction between acute versus chronic effects of alcohol on TTP by fitting three different models: acute consumption patterns, chronic measures of alcohol, or both acute and chronic alcohol exposures, and use fit statistics to assess the best fitting model. We will also model the use of alcohol during the course of the study as a function of TB disease severity using a repeated measures model based on self-reported alcohol usage at each time point. All models will assess goodness-of-fit. Analyses will be performed in R (r-project.org) and SAS (SAS Institute, Cary, NC).

For the second objective, we will investigate the effect of problem alcohol use on the PK measures peak serum concentration (Cmax) and area under the curve (AUC) and the effect on sterilizing activity, with adjustment for drug concentrations and mycobacterial drug minimal inhibitory concentration (MIC). We will identify other potential modifiers of the alcohol-drug concentration-sterilization relationship, including BMI, gender, and age, and use these findings to build a prediction model of who is at highest risk of low PK with delayed PD. We will also assess how these other modifiers explain variability in PK/PD seen in the control, non-problem alcohol population.

#### Innovative modeling techniques

In order to incorporate the variability of *Mycobacterium tuberculosis* drug susceptibility and the complexity of the multidrug pharmacokinetic relationship, we will model the nonlinear effects of TB drug exposure together with MIC and the interaction between drug exposures, on sterilizing activity [36] in participants exposed to alcohol compared to those without alcohol exposure. This nonlinear semi-mechanistic model uses TTP data in sputum liquid cultures at 12 weeks as a surrogate of bacillary burden to describe the biexponential decline in bacillary load using the α- and β-slopes.

The slowly declining *M. tuberculosis* subpopulation is thought to represent ‘persister’ bacilli and their rate of kill is thought to correspond to sterilizing activity of the regimen, defined as β-slope. Using a multivariate adaptive regression splines (MARS) analysis, individual estimates of β-slope will be generated by the model for evaluation of the effects of drug exposure on sterilizing activity. As drug interactions can be additive, synergistic or antagonistic, by using this non-parametric regression data analysis technique, we will be able to combine recursive partitioning into sub regions of interest with fitting of splines to variables to determine relationships within the sub-regions in the dataset. Potential predictors of the primary outcome (i.e. the β- slope) included in the MARS analysis will be problem alcohol use (versus non), PK measurements, individual drug MICs, the percentage of the 24-h dosing interval that concentration exceeded MIC and any other variables found to significantly predict final outcomes in the initial analysis. By confirming preliminary findings of variability of specific drug PK among participants and the association with 12-week culture conversion and rate of decline in slowly dividing *M. tuberculosis* subpopulations (β-slope or sterilizing activity) in the PD model, we will be able to evaluate if drug dosing in participants using alcohol requires more intensive monitoring and if dose regimens should specifically be adjusted for alcohol.

Finally, we will use mediation analysis methods to estimate both the direct and indirect effects of alcohol on TB treatment outcomes in the presence of potentially mediating effects of adherence and medication PK [37].

### Project status

Recruitment began in May 2017 and is expected to complete by October 2020.

## Discussion

TRUST will define the physiologic role of alcohol use on TB treatment outcomes independent of its behavioral effects, thus introducing a new paradigm of how to craft interventions to improve outcomes for this population. The anticipated challenges inherent in studying this at-risk population are high participant attrition and low enrollment; however, we have built a system robust against attrition through comprehensive collection of information on how to locate and contact participants, active tracking and engagement of participants between appointments using DOT workers and mobile technology, and consistent provision of reminders and reimbursement to minimize loss to follow up.

Through TRUST’s novel approach of using repeated validated alcohol measures along with collection of comprehensive treatment adherence information, we aim to determine the contribution of acute versus chronic alcohol use on TB treatment response, leading to an improved understanding of how long alcohol effects persist and inform tailored treatment for patient populations with this modifiable TB risk factor.

Furthermore, though modeling, TRUST aims to change the approach to optimizing drug dosing for TB, with the potential to improve therapeutics for patients with co-morbid problem alcohol use. For instance, higher doses of isoniazid and rifampin have been demonstrated to be well-tolerated in clinical trials [38, 39], so should we find either consistently low PK or delayed effectiveness of these two drugs among alcohol users, empiric increased dosing of either drug could be further studied. Alternatively, if one of the first line drugs is found to consistently underperform, consideration could be given to substituting an alternative TB medication such as a fluoroquinolone; this substitution has been shown to be non-inferior to standard therapy [40]. The findings from the TRUST study may inform blanket regimen modification for problem alcohol users, or tailored modifications based on a risk profile of those at risk of low PK or delayed PD based on our prediction model.

Our prospective investigation of the relation between problem alcohol use and TB treatment will provide the basis for identifying novel interventions to improve cure rates in TB patients with co-morbid problem alcohol use. TRUST will inform new strategies for addressing the complex interactions between these diseases, which could include risk profiling to identify patients with problem alcohol use, innovative medication dosing, medication substitutions, changes in adherence monitoring, and extending length of treatment.

## Additional Files

Additional File 1: TRUST Protocol. This document provides the full protocol for the TRUST Study as of August 2017. (PDF 744 kb)

Additional File 2: Ethical approval reference numbers. This document contains a list of the specific names and reference numbers for all ethical bodies that approved the study in the various participating and involved centers. (DOCX 13 kb)

## Abbreviations

ALT: Alanine transaminase
AST: Aspartate transaminase
AUC: Area under the curve
CBC: Complete blood count
Cmax: Peak serum concentration
Cr: Creatinine
DOT: Directly observed therapy
HbA1c: Hemoglobin A1C
INR: International normalized ratio
MARS: Multivariate adaptive regression splines
MIC: Minimal inhibitory concentration
PD: Pharmacodynamics
PK: Pharmacokinetics
PT: Prothrombin time
TB: Tuberculosis
TRUST: Tuberculosis Treatment and Alcohol Use Study
TTP: Time to positivity
