# Supplementary material for: Impact of alcohol consumption on tuberculosis treatment outcomes: a prospective longitudinal cohort study protocol
Source: BMC Infect Dis. 2018 Sep 29;18:488. doi: 10.1186/s12879-018-3396-y (PMC6162918; doi:10.1186/s12879-018-3396-y)
Supplement: Supplementary file 1 — TRUST Protocol. This document provides the full protocol for the TRUST Study as of August 2017. (PDF 744 kb) [file 12879_2018_3396_MOESM1_ESM.pdf]

The Impact of Alcohol Consumption on TB Treatment Outcomes

Sponsored by:
National Institute of Allergy and Infectious Diseases (NIAID)

Principal US Investigator: Karen R. Jacobson, MD, MPH
Assistant Professor of Medicine
Section of Infectious Diseases
Boston University School of Medicine

Principal South African Investigator: Bronwyn Myers, Ph.D.
Chief Specialist Scientist
Alcohol, Tobacco and Other Drug Research Unit
South African Medical Research Council

DMID Protocol Number:
16-0043

Keywords:
Tuberculosis, alcohol, treatment outcomes

Version Number: 3.2

29 August 2017

Statement of Compliance

This study will be conducted in compliance with the protocol, International Conference on Harmonisation Good Clinical Practices E6 (ICH-GCP) and the applicable regulatory requirements.

- U.S. Code of Federal Regulations applicable to clinical studies (45 CFR 46 – Protection of Human Subjects).
- Completion and certification of the Human Subjects Protection Training at all participating institutions or offered equivalent that meet the requirements of NIH Policy "*Required Education In the Protection Of Human Research Participants*" (Notice OD-00-039), Revision Date: August 25, 2000.
- ICMR Ethical Guidelines for Biomedical Research on Human Subjects and Guidelines for Good Clinical Laboratory Practices.

SIGNATURE PAGE

The signature below constitutes the approval of this protocol and the attachments, and provides the necessary assurances that this trial will be conducted according to all stipulations of the protocol, including all statements regarding confidentiality, and according to local legal and regulatory requirements and applicable US federal regulations and ICH guidelines.

We, Drs. Jacobson and Myers, have read the Department of Health: Ethics in health research: principles, processes and structures, second edition, 2015, the Guidelines for Good Practice in the Conduct of Clinical Trials with Human Participants in South Africa, Second Edition, 2006, Department of Health, Pretoria, South Africa (where applicable), and the Declaration of Helsinki (2013) and have prepared this proposal with due cognisance of its content. Furthermore we will adhere to the principles expressed when conducting this proposed research project.

Table of Contents

page
Statement of Compliance i
Signature Page ii
List of Abbreviations v
Protocol Summary vi

1 Key Roles 1
2 Background Information and Scientific Rationale 3
2.1 Background Information 3
2.2 Rationale 3
2.3 Potential Risks and Benefits 6
2.3.1 Potential Risks 6
2.3.2 Known Potential Benefits 8
3 Objectives 9
4 Study Design 10
5 Study Population 11
5.1 Selection of the Study Population 11
5.2 Inclusion/Exclusion Criteria 13
6 Study Procedures/Evaluations 15
6.1 Study Procedures 15
6.2 Laboratory Evaluations 17
6.2.1 Laboratory Evaluations/Assays 17
6.2.2 Special Assays or Procedures 18
6.2.3 Specimen Shipping 18
7 Study Schedule 19
7.1 Pre-Screening 19
7.2 Screening 19
7.3 Enrollment/Baseline Visit (Treatment initiation visit) 19
7.4 Weekly Visits (Treatment Weeks 1-12) 20
7.5 Daily Assessments (Months 1-6) 20
7.6 Single PK/PD Visit (In Treatment Week 4 – Treatment Week 8 window) 20
7.7 Monthly Follow-Up Visits (Treatment Month 1-6) 20
7.8 Post-Treatment Follow-Up (Months 3, 6, 9, 12 post treatment) 21
7.9 Criteria for Discontinuation or Withdrawal of a Participant (or a Cohort), if applicable 21
8 Assessment of Outcome Measures 22
8.1 Specification of the Appropriate Outcome Measures 22
8.1.1 Primary Outcome Measures 22
8.1.2 Secondary Outcome Measures 22
9 Safety assessment and reporting 23

Table of Contents - *continued*

page
9.1 Definition of Adverse Event (AE) ... 23
9.2 Definition of Serious Adverse Event (SAE) ... 23
9.3 Reporting Procedures ... 24
9.3.1 Serious Adverse Event Detection and Reporting ... 24
9.3.2 Procedures to be Followed in the Event of Abnormal Laboratory Test Values or Abnormal Clinical Findings ... 24
9.3.3 Type and Duration of the Follow-up of Participants After Adverse Events ... 25
10 Clinical Monitoring Structure ... 26
10.1 Site Monitoring Plan ... 26
11 Statistical Considerations ... 27
11.1 Study Outcome Measures ... 27
11.2 Sample Size Considerations ... 28
11.3 Participant Enrollment and Follow-Up ... 28
11.4 Analysis Plan ... 28
12 Access to Source Data/Documents ... 30
13 Quality Control and Quality Assurance ... 31
14 Ethics/Protection of Human Participants ... 32
14.1 Ethical Considerations ... 32
14.2 Declaration of Helsinki ... 32
14.3 Institutional Review Board ... 32
14.4 Informed Consent Process ... 32
14.4.1 Informed Consent Process ... 33
14.5 Exclusion of Women, Minorities, and Children (Special Populations) ... 33
14.6 Participant Confidentiality ... 34
14.7 Future Use of Stored Specimens ... 34
15 Data Handling and Record Keeping ... 35
15.1 Data Management Responsibilities ... 35
15.2 Data Capture Methods ... 35
15.3 Types of Data ... 36
15.4 Timing/Reports ... 36
15.5 Study Records Retention ... 36
15.6 Protocol Deviations ... 36
16 Publication Policy ... 37
17 Literature References ... 38

SUPPLEMENTS/APPENDICES
A: Study Schedule

List of Abbreviations

AE	Adverse Event
AFB	Acid Fast Bacilli
ALT	Alanine Transaminase
AST	Aspartate Transaminase
AUC	Area Under the Curve
AUDIT	Alcohol Use Disorders Identification Test
BMC	Boston Medical Center
BUSM	Boston University School of Medicine
CBC	Complete Blood Count
CDC	Community Day Centre
CES-D	Center for Epidemiologic Studies Depression Scale
CFR	Code of Federal Regulations
CIOMS	Council for International Organizations of Medical Sciences
CRF	Case Report Form
CXR	Chest X-Ray
DMID	Division of Microbiology and Infectious Diseases
DOTS	Directly observed therapy, short course
DUDIT	Drug Use Disorders Identification Test
EMB	Ethambutol
FTND	Fagerstrom Test for Nicotine Dependence
FWA	Federal-Wide Assurance
GCP	Good Clinical Practice
HIPAA	Health Insurance Portability and Accountability Act
HIV	Human Immunodeficiency Virus
HR	Hazard Ratio
ICF	Informed Consent Form
ICH	International Conference on Harmonisation
INH	Isoniazid
IRB	Institutional Review Board
MDR TB	Multidrug resistant tuberculosis
MGIT	Mycobacteria Growth Indicator Tube
MIC	Minimum inhibitory concentration
MOP	Manual of Procedures
MTB	*Mycobacterium tuberculosis*
N	Number (typically refers to participants)
NIAID	National Institute of Allergy and Infectious Diseases, NIH, DHHS
NIH	National Institutes of Health
OCRA	Office of Clinical Research Affairs, DMID, NIAID, NIH, DHHS
OHRP	Office for Human Research Protections
ORA	Office of Regulatory Affairs, DMID, NIAID, NIH, DHHS

List of Abbreviations - *continued*

PD	Pharmacodynamics
PETH	Phosphatidylethanol
PI	Principal Investigator
PK	Pharmacokinetics
PZA	Pyrazinamide
RIF	Rifampicin
REC	Research Ethics Committee
QA	Quality Assurance
QC	Quality Control
SAE	Serious Adverse Event
SAMRC	South Africa Medical Research Council
SOP	Standard Operating Procedure
TB	Tuberculosis
TLFB	Timeline Follow Back
TTP	Time to positivity
UCT	University of Cape Town
WHO	World Health Organization

Protocol Summary

Title: The impact of alcohol consumption on TB treatment outcomes

Population: 438 participants (male and female, ages 15 years and older, HIV infected and uninfected) initiating treatment for drug susceptible TB disease in the Worcester CDC in Worcester, South Africa. We expect 30% to be HIV positive.

Number of Sites:

- Worcester Community Day Centre (CDC) and Brewelskloof Hospital, Worcester, South Africa
- Boston Medical Center/Boston University School of Medicine, Boston, MA
- South Africa Medical Research Council (SAMRC), Tygerberg, South Africa
- University of Cape Town, Cape Town, South Africa

Study Duration: 6 years (including time for manuscript preparation)
Budget duration: 5 years

Participant Duration: 18 months

Objectives:

Aim 1. To (i) examine the associations between problem alcohol use and TB treatment outcomes, and (ii) demonstrate that these associations persist independent of adherence to TB treatment among HIV-infected and uninfected participants.

Hypothesis 1a: Poor TB treatment outcomes, measured as delayed time-to-culture conversion, are associated with problem alcohol use after controlling for non-adherence.
Hypothesis 1b: End organ damage due to chronic alcohol use, rather than effects of acute ingestion, will be the strongest predictor of poor treatment outcomes independent of adherence.

Aim 2. To evaluate the effect of problem alcohol use on the PK/PD of TB drugs among HIV uninfected participants.

Hypothesis 2a: Persons with problem alcohol use will have altered peak drug concentration (Cmax) and area under the curve (AUC) due to effects on drug absorption and metabolism.
Hypothesis 2b: These PK changes will be associated with delayed culture conversion and higher treatment failure/relapse rates; and/or with increased toxicity resulting in regimen changes or death.

Overview of Study Design:

We will recruit microbiologically-confirmed, pulmonary TB patients in Worcester, South Africa, and follow them over an 18-month period. Patients will complete an interviewer-administered questionnaire on their alcohol use and other health-related behaviors, including tobacco and illicit drug use, and their recent alcohol use will be confirmed using a biomarker (phosphatidylethanol). We will collect chest radiographs, sputum smears and culture, and blood samples to compare the biology of treatment response in patients with and without problem alcohol use. During the 6-month treatment period, we will use smart mobile-phone technology to document daily drug adherence by trained community workers and will collect serial measures of alcohol intake and serial sputa isolates to assess treatment response, and record TB drug side effects. In addition, we will perform intensive PK/PD studies of isoniazid, rifampin, ethambutol, and pyrazinamide in 200 HIV-seronegative patients. We will follow the full cohort for 12 months post-treatment to examine long-term TB outcomes, including relapse and death.

<u>Schematic of Study Design</u>:

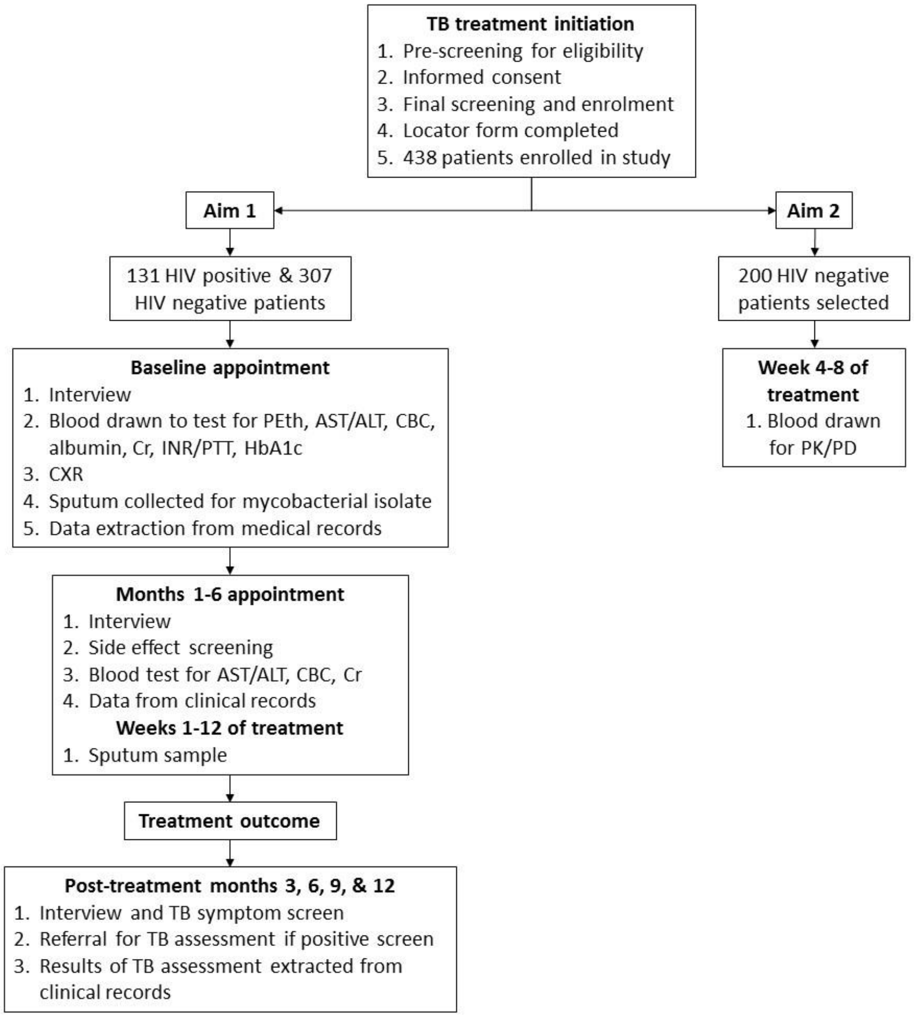

1 KEY ROLES

Study Sites:

Boston University (BU) and Boston Medical Center (BMC)
801 Massachusetts Avenue
Boston, MA 02118, USA

Medical Research Council of South Africa (SAMRC)
Alcohol, tobacco and Other Drug Research Unit
Tyberberg, South Africa 7505

Stellenbosch University (SU)
DST/NRF Centre of Excellence for Biomedical Tuberculosis Research
US/SAMRC Centre for Tuberculosis Research
Tygerberg, South Africa 7505

University of Cape Town (UCT)
Division of Clinical Pharmacology
Observatory, Cape Town, South Africa 7925

Worcester Community Day Centre (CDC)
Sugget Street
Worcester, South Africa 6850

Brewelskloof Hospital (BKH)
Haarlem Street
Worcester, South Africa 6850

Ukwanda Rural Clinical School – Stellenbosch University (Ukwanda)
Durban Street
Worcester, South Africa 6850

Project Roles:

Study Investigator	Institution	Role
**Karen Jacobson**	Boston Medical Center	BMC PI
**Bronwyn Myers**	SAMRC	Local/Subcontract PI
**C. Robert Horsburgh**	Boston University	Subcontract PI
**Helen McIlleron**	University of Cape Town	Subcontract PI

**Robin Warren**	Stellenbosch University	Subcontract PI
**Charles Parry**	SAMRC	SAMRC Co-Investigator
**Laura White**	Boston University	BU Biostatistician

2 BACKGROUND INFORMATION AND SCIENTIFIC RATIONALE

2.1 Background Information

An urgent need exists globally to identify modifiable drivers of poor TB treatment outcomes. TB is the leading cause of death due to an infectious disease globally. Despite recent declines in TB incidence, approximately 8.6 million new TB cases occurred in 2012.[1] The slight global decline in incidence conceals regional variations; particularly the African and European regions did not halve 1990 mortality levels by 2015. South Africa is the third highest TB burden country, with an estimated TB incidence of 1,003 cases per 100,000 population, and has failed to achieve 85% treatment success for smear positive pulmonary TB cases.[2] In South Africa's Western Cape Province, where this study will be based, TB is the leading cause of natural death.[3] In Cape Town, approximately 15% of TB cases result in treatment failure and 26% of the total TB burden are re-treatment cases, which is higher than elsewhere on the African continent.[4] Understanding drivers of TB vulnerability and poor treatment response, both at the individual and population level, is essential for TB control – even more so with the recent rise of drug resistant TB strains.

Problem alcohol use in vulnerable populations is a key driver of poor TB treatment response, both globally and in South Africa. In high-income countries, non-communicable diseases, including diabetes and alcohol use disorders, are thought to be more significant causes of immunological impairment in TB patients than HIV due to their greater prevalence.[5] In high-HIV burden countries like South Africa, excess alcohol consumption may synergistically with HIV weaken the immune system, leading to more progression to active TB disease and poorer TB treatment response.[6] A report from the United States National Tuberculosis Surveillance System, 1997-2012, found that 15% of all TB cases and 25% of US-born TB cases had documented excess alcohol use.[7] Patients with excess alcohol use were more likely to have smear positive disease, delays in culture conversion, and higher mortality while on TB treatment. Better understanding of the relationship between alcohol and TB is needed to guide treatment of these patients in order to both improve individual outcomes and decrease transmission to others. Problem alcohol use, defined as a volume or pattern of alcohol consumption that results in adverse health events, is on the increase in low- and middle income countries, including South Africa.[8] Overall, 10% of TB deaths globally have been attributed to alcohol as a risk factor.[9] This number is estimated to be even higher in South Africa, with 23% of male and 7% of female TB deaths attributable to alcohol.[8] Similarly in China and India, the two highest burden TB countries, 11-13% of male TB deaths are attributable to alcohol,[9] making findings from this study potentially applicable to many other high TB burden settings globally.

2.2 Rationale

Alcohol's large attributable risk for TB deaths in South Africa is likely due in part to South Africa's having one of the highest levels of alcohol consumption *per drinker* globally and one of the highest rates of heavy episodic drinking.[8] The nationally representative South African Stress and Health Study found that 20% of adults in the Western Cape Province were likely to develop

a substance use disorder in their lifetime, with alcohol being the most common abused substance.[10] Problem alcohol use is especially prevalent in the rural farming regions of this province, where this study will be conducted. Upwards of one-third to close to 60% of men and one-third to close to 50% of women in these communities self-report heavy episodic drinking and screen positive for problem drinking using standardized screening tools.[11-13]

There is a critical need for more effective TB treatment strategies for patients with a history of problem alcohol use. Alcohol use is greatly overrepresented among active TB patients. Screening patients with problem alcohol use in an urban setting for TB infection revealed rates of active TB that were 28-fold greater than those of age-matched residents.[14] A meta-analysis reported a three-fold increased risk of active TB in individuals who consume more than 40g ethanol/day or who have an alcohol use disorder.[15] In addition, retrospective studies from many countries have consistently reported that patients with problem alcohol use have worse TB treatment outcomes, including higher rates of treatment failure (defined as persistently smear positive after 4 months of therapy), relapse, and death compared to patients who do not consume alcohol.[16-20] Alcoholic patients in Brazil had a 2.8-fold increased odds of treatment failure compared to nonalcoholics.[17] Patients admitted to hospitals in Russia for treatment of chronic or relapsing TB had 3 times higher rates of alcohol use disorders compared to patients newly infected with TB.[21] A 2.7-fold increased risk of death during TB treatment was reported in Russia for patients with problem alcohol use compared to those without, and there was a 4.4-fold increased risk of death during TB treatment in US patients with reports of excessive alcohol use compared to those without.[22,23] All of these studies demonstrate worse treatment outcomes in patients with problem alcohol use, supporting the need for more effective, safer, and better tolerated TB treatment strategies for this vulnerable population.

A major knowledge gap is the degree to which poor treatment outcomes in alcohol-abusing patients are due to noncompliance alone. Problem alcohol use impacts on retention in care and adherence to daily TB treatment. Poor medication adherence and increased default from TB care have been documented for patients consuming alcohol regularly in several countries, including Russia, Brazil, India, Estonia, and in PI Jacobson's studies of multidrug resistant (MDR) TB patients in Worcester, South Africa, where patients reporting alcohol use had a 2.1-fold higher rate of default compared to patients with no alcohol use history.[18,24-27] A systematic review found a pooled odds ratio of 3.0 (95% CI: 1.8-5.0) for alcohol dependence as a default predictor.[9] A study in India, in which there was a high prevalence of diabetes, low body mass index (BMI), and cavitary disease, reported that drinking alcohol during treatment for MDR TB was the only factor independently associated with unsuccessful outcomes.[17] Although those who drank during treatment missed more doses than those who did not, which likely contributed to the poor outcomes, it is noteworthy that some alcohol-consuming patients who were adherent still had unsuccessful treatment. Yet there has been no research to identify reasons (beyond adherence) for these poorer outcomes among patients with problem alcohol use. A key barrier to understanding the persistent biologic effect of alcohol on TB disease is inadequate data on adherence, including detailed data on daily adherence (or number of missed doses of medication).[18,22,24,25] Research combining better approaches to alcohol ascertainment and adherence monitoring is needed to advance understanding of the pathways by which alcohol use and TB disease interact.

Recent studies suggest that much of the increased rate of treatment failure in TB patients may be due to microbiologic failures not directly attributable to nonadherence. In recent hollow-fiber studies, in which degrees of noncompliance were modelled, poor adherence was associated with microbiologic failure only when nonadherence exceeded 60%, and acquired drug resistance, defined as loss of susceptibility to one of the TB drugs, never occurred with nonadherence to a 4-drug regimen.[28] Similarly, a meta-analysis concluded that studies with lower default rates did not differ significantly in microbiologic failure, acquired drug resistance, or relapse compared to studies with higher default rates.[29] The authors theorized that microbiologic failures emerge when patients are consistently under-dosed or due to PK and microbial variability. Thus, although it is important to develop systems that will help patients who use alcohol to stay in treatment, the higher rates of poor microbiological outcomes may also be due to the impact of alcohol use on PK and bacterial susceptibility or other mechanisms driving treatment response, that need to be better understood.

Evaluation of sources of PK variation can facilitate optimization of TB treatment regimens by identifying avoidable sources of variation and risk factors for low or high drug concentrations in patients. Several studies have shown the importance of optimal TB drug concentrations on the rate of kill of MTB in hollow fiber systems, in animal models of TB, and in patients.[30-32] Clinical studies have also shown low drug concentrations to be associated with poorer outcomes in patients receiving combination therapy.[33-35] In one study, patients with problem alcohol use had a significant decrease in the absorption and increase in the metabolism of the first-line TB drug isoniazid (INH), which led to lower maximum concentrations of the drug and a shorter half-life.[36] Researchers have reported increased acetylation in rat liver cells in the presence of ethanol, leading to speculation that alcohol enhances the activity of N-acetyltransferase, which also catalyzes the metabolism of INH, potentially leading to an increased metabolism rate.[37] Problem alcohol use has also been associated with acquisition of drug resistant TB (primarily INH and rifampicin (RIF) resistance), which may reflect a failure to attain optimal drug levels. In Indonesia, MDR TB was found to be associated with excessive alcohol use, and TB patients admitted to hospitals in Russia were found to have an 8-fold increased likelihood of TB drug resistance if they had a history of an alcohol use disorder.[21,38] A detailed analysis of the PK of all 4 TB drugs in patients with problem alcohol use and their impact on PD will allow for improved understanding of which TB drugs are most affected in this population. This information will allow for more appropriate, increased dosing of specific drugs, drug substitutions for individual drugs that are consistently performing poorly, and potential extension of the intensive or continuation phases of therapy in this at-risk population.

Evidence supports the additional biologic impact of chronic alcohol use on TB outcomes. Attention has been focused on the need to stop or decrease acute alcohol ingestion during TB treatment, but the lower rates of cure may also be due to chronic sequelae of years of alcohol use. Alcohol ingestion interferes with anatomic barriers that protect the lung from infection, impairs the actions of cytokines such as tumor necrosis factor alpha, and inhibits the expression of growth factors such as lung granulocyte colony stimulating factor.[39] Functional activities of macrophages, including mobilization, adherence, phagocytosis, superoxide production, and microbicidal activity, are inhibited by alcohol.[39] Alcohol-induced deficits in alveolar macrophage function diminish the capacity of these cells to eliminate invading pathogens. Harmful effects of chronic alcohol use may be of particular importance in TB where more than 90% of inhaled mycobacteria are normally destroyed by alveolar macrophages.[400] Bacilli not killed by alveolar

macrophages survive and proliferate intracellularly. Studies have shown that exposure to alcohol enhances intracellular growth of mycobacteria in human macrophages.[41,42] These immune deficits could also be exacerbated by concomitant nutritional deficiencies or tobacco use that are often associated with chronic alcohol use.[43,44] Chronic alcohol exposure may suppress cytokine production, which has an essential role in cellular communication, activation, proliferation, migration, and regulating inflammation and other healing mechanisms.[45] Utilizing a pulmonary route for MTB infection in a mouse model, researchers found fewer CD4+ cells in the lungs of mice chronically exposed to alcohol, one likely reason for their poorer containment of the mycobacterial challenge compared to non-alcohol consuming mice.[46] This inability to increase the CD4+ population in response to pulmonary infections is at least partially due to diminished IL-2 production and lymphocyte proliferation in regional lymph nodes. Additionally, granuloma formation was abnormal in the alcohol-consuming mice. These authors also found a diminished capability of the CD4+ cells to secrete IFN-γ when stimulated with MTB-infected macrophages. These findings suggest mechanisms that may be responsible for compromise of the ability to contain and effectively clear TB infection in patients with chronic alcohol exposure, independent of medication adherence.

2.3 Potential Risks and Benefits

2.3.1 Potential Risks

Risks specific to study participation are low. The study procedures that can cause physical harm are:

- Chest x-ray: Standard chest x-rays will be performed if not already done by the South Africa TB program (chest x-rays are standard on TB patients at diagnosis in the United States). There is always a slight risk of damage to cells or tissue from being exposed to any radiation, including the low levels of radiation used for x-rays. However, the risk of damage from the standard chest X-ray is usually very low compared with the potential benefits of the test. Given the minor risk associated with radiation exposure, we will not perform chest X-rays on any women who are pregnant.

- Blood collection through venipuncture: There is very little risk associated with venipuncture. Some patients may experience minor discomfort, minimal bleeding, a bruise or swelling at the site of puncture. In rare cases patients may experience bleeding at the site of puncture, fainting or feeling light-headed, hematoma, localized infection, multiple punctures to locate veins. Any area where the skin anesthetic will be applied may become red and/or irritated. There are no known alternatives to venipuncture. Separate sterile equipment will be used on all participants. Emergency services will be available in the event of adverse events. The study nurses will be trained and experienced in phlebotomy prior to implementation of the study.

Strict airborne respiratory precautions will be taken when patients are providing sputum samples to prevent secondary exposure of clinical staff to infectious droplets.

Adequacy of Protection against Risks

Recruitment and Informed Consent: Recruitment will be open to all eligible participants, and every effort will be made to enroll a representative and unbiased sample of participants. Potential participants will be provided with an accurate and fair description of the risks or discomforts and the anticipated benefits of the research study, as outlined in 45 CFR 46, subpart A, as well as the International Conference on Harmonization – Good Clinical Practice (ICH-GCP) guidelines and the South African guidelines for Good Clinical Practice (GCP), second edition. Eligible participants identified through a structured pre-screening process who are recruited for screening will meet with a trained research assistant who will describe the purpose, goals, investigator and participant responsibilities and conduct of the study. The description will emphasize the design of the study, objectives and participant expectations, the voluntary participation, and confidentiality. It will be made clear that participation in this study does not affect the routine healthcare they will receive. Only participants with signed or marked consents will be enrolled. Separate consent will be obtained from parent/legal guardian for children under the age of 18 in addition to consent from minor children age 15-17 years.

Protection against Risk:

Confidentiality: Using study identification numbers as identifiers on case report forms, clinical specimens, and computerized databases containing study information will minimize risks to breach of confidentiality. Personal and medical information regarding study participants will not be released to anyone other than authorized study personnel, the National Institutes of Health – United States and/or its designee, the IRB at BMC/BU, or the applicable Research Ethics Committee(s) (REC) at the Medical Research Council of South Africa or University of Cape Town, without written permission of the participant. Study records will be secured in locked file cabinets at the South African site and electronic files will be secured on password protected computers. The study database will be maintained on REDCap. This database will be electronically encrypted using secure socket layering (SSL) encryption technology and will reside on a secure, password and firewall protected server at Boston University Medical Center (BUMC). All data stored on the database will only be identifiable through a study participant ID number. For programmatic purposes, participants will be entered into the mobile DOTS system by both participant ID and first name and first initial of surname. The first name and first initials of the surname will be entered into the DOTS system so that DOTS workers can address the participant by their name, and also ask to speak to the participant by name (rather than study number) when calling them. Communications with the sites (clinical and laboratory) will use only Study ID number to identify participant records. All study documents, including patient records, laboratory data, and correspondence will be retained by the investigator for at least five years after the last participant has reached the final study data point.

Safety: Routine procedures will be performed by experienced, trained and qualified nursing staff employed by this study. Study-related questionnaires will be administered in a culturally sensitive manner by trained research assistants who are fluent in both English and Afrikaans languages. Our preliminary work in the Worcester region has highlighted that all patients receiving care at the Worcester CDC will be Afrikaans or English speaking. TB treatment staff employed at these facilities are also Afrikaans and English speaking, with patients who are only comfortable in isiXhosa being treated at the nearby Empilsweni clinic. Necessary training from an experienced investigator will be conducted with field staff. Participants will receive TB

treatment as standard of care in this region. Patients with MDR TB, although excluded from this study, will also continue to receive standard care for their MDR TB disease. Potential psychological harm associated with alcohol abuse assessments will be minimized through the use of well-trained health educators, the provision of a health information card on ways of minimising alcohol harms and the option of referral to a local alcohol treatment center or the clinic's social worker for further assessment and referral. Alcohol use among minors will not be reported to parents as this may increase risk of harms. Pregnant women will not be included in this study at enrollment and, therefore, will not be exposed to the additional risk associated with chest x-rays. If a woman becomes pregnant during the study period she will not be censured as there is no additional risk to her being in this cohort after the initial x-ray.

Adverse Event Monitoring: Serious adverse events associated with the conduct of the study will be reported to the Ethics Committees in Boston and South Africa and the NIH in accordance with DHHS and international guidelines within three days of awareness of their occurrence. Due to the laboratory/epidemiologic focus of the study, few serious adverse events are anticipated in this study.

2.3.2 Known Potential Benefits

Potential benefits of the proposed research to the study population include:

- Participants will be followed with intensive monitoring for adherence measures which has the potential to decrease default from care.
- Participants will receive close monitoring for 12 months after TB treatment completion for disease relapse or reinfection. Early recognition of relapse will allow for appropriate and timely treatment that may prevent further transmission to close contacts.
- Participants who report substance use will be offered a referral to a local substance abuse treatment service and/or the social worker associated with the TB clinic. Uptake of this referral will be voluntary. Monitoring the uptake of these referrals is beyond the scope of this cohort study. To minimise risk of harms associated with continued alcohol use while receiving TB treatment, all patients will be advised not to drink while on TB treatment and will receive an alcohol information handout that will outline the potential risks of alcohol use while receiving TB treatment.

3 OBJECTIVES

This prospective investigation of the relation between problem alcohol use and TB treatment will provide the basis for identifying novel interventions to improve cure rates in TB patients with co-morbid problem alcohol use. New strategies for addressing the complex interactions between these diseases could include risk profiling to identify patients with problem alcohol use, innovative medication dosing, medication substitutions, changes in adherence monitoring, and extending length of treatment.

Aim 1: To (i) examine the associations between problem alcohol use and TB treatment outcomes, and (ii) demonstrate that these associations persist independent of adherence to TB treatment among HIV infected and uninfected participants.

Aim 2: To evaluate the effect of problem alcohol use on the PK/PD of TB drugs among HIV-uninfected participants.

4 STUDY DESIGN

We will employ a longitudinal, repeated measures, observational cohort design (see Figure: Schematic Study Design above).

For Aim 1, we will recruit 438 patients who are initiating drug susceptible pulmonary TB treatment according to South African treatment guidelines. Immediately following study enrollment, participants will complete an interviewer-administered questionnaire, a urine drug test will be administered, and blood will be drawn for biologic tests, including a dried blood spot for phosphatidylethanol (PEth) alcohol biomarker testing. Results from the baseline blood tests will be provided back to TB Program. We will collect sputum for mycobacterial culture and we will extract clinical data on previous TB disease, Xpert MTB/RIF result, HIV status, and general physical health from treatment records. Minimum inhibitory concentrations (MICs) for INH, RIF, EMB, and PZA (the four drugs in drug susceptible TB treatment) will be determined on baseline sputum isolates. MICs for INH will be performed while the participant is still on the intensive phase of treatment and results will be provided back to TB Program. Participants will then continue on standard of care delivered by the national TB program through their clinic: participants will receive standard TB therapy; receive monthly follow up appointments at the clinic where their national TB program DOTS worker cards will be checked; produce sputum samples to check for treatment response (AFB smear conversion) at 2 and 5 months; and be verified at 6 months as cured or failed therapy.

In addition to this standard of care, for Aim 1 of the research study, the research study DOTS workers will observe and document TB medication ingestion 5 days/week and have participants self-report weekend compliance. Participants will provide weekly sputum samples during the first 12 treatment weeks to measure time to positivity (TTP) to assess rate of sterilization/culture conversion as well as smear conversion. We will conduct follow up interviews every month for the 6 treatment months in which a brief interviewer-administered questionnaire (alcohol use and TB medication side effects) will be done and data on participants' weight and AFB smear when available (2 and 5 months) will be extracted from their medical records. Participants will also provide a sputum specimen at their 5 month study visit to validate smear results from the medical record. If there is a positive side effect screen at any appointment, blood will be drawn to test for complete blood count (CBC), alanine transaminase (ALT), aspartate transaminase (AST) and creatinine (Cr). These lab results will be given to TB Program. For the research study, participants will also be interviewed at 3, 6, 9, 12 months post-treatment and screened for any re-occurrence of TB symptoms. Participants with a positive TB symptom screen will be referred back to the clinic for microbiologic assessment, and we will extract the results of these assessments from their clinical records. In total, patients will be enrolled in the study for 18 months.

For Aim 2, we will enroll a subgroup of 200 HIV-negative patients from our original cohort to participate in a single 8-hour TB drug PK testing during treatment weeks 4-8. We will follow standard procedures for collecting, storing and transporting samples, successfully used in our previous studies.[47-49]

5 STUDY POPULATION

We will recruit patients from the Worcester Community Day Centre (CDC), a clinic in Worcester, the main town of the Breede Valley municipality in the Western Cape Province, South Africa. TB is endemic in this region, with an incidence of 1000 cases/100,000 population, and the problem alcohol use rate is exceedingly high.[4,50] This clinic provides free TB treatment services to patients residing within its catchment area, which includes the town of Worcester and the surrounding low socioeconomic status (SES) communities that are inhabited primarily by "Coloured" people (of mixed race ancestry), a population of ~150,000. In 2013, the TB clinic in the Worcester CDC started 664 patients on TB treatment, 75% of whom were >15 years (from the Electronic Tuberculosis Record or ETR.net, South Africa). 92% of adult TB patients had a documented HIV test and 30% were positive. Prior to initiating treatment, patients with suspected TB are screened for active TB disease by a symptom-based evaluation and microbiologic assay (Xpert MTB/RIF, AFB smears, and mycobacterial cultures). Patients are asked to return to the clinic 2-3 days later to receive results and initiate treatment if they test positive for active TB. Study participants will receive standard drug susceptible TB therapy from the clinic, which consists of 6 months of antibiotic treatment: a 2-month intensive phase of 4 drugs (INH, RIF, EMB, PZA) and then a 4-month continuation phase with 2 drugs (INH, RIF). Patients are given a 1-month take home supply of medication and return on a monthly basis to the clinic for monitoring and to receive their next month's medication supply until the 6-month treatment period has been completed. Sputum samples are collected to document microbiologic cure at the 2- and 5-month treatment visits. In our preliminary work, we found that almost all patients attending this clinic speak Afrikaans and/or English (as do the clinic staff); as a result assessments will be offered in these languages. Patients who are isiXhosa speaking and who are not comfortable in speaking Afrikaans or English are generally treated at Empilsweni clinic and therefore would not be eligible for this study. In the rare event that a potential study participant speaks neither of these languages, they will not be enrolled in the study. Native Afrikaans speakers will translate the interviewer-administered questionnaires into Afrikaans using standard forward and back-translation techniques used in other studies.[51]

5.1 Selection of the Study Population

The participant population for this study will include all patients being treated for active pulmonary TB disease at Worcester CDC in Worcester, South Africa. Enrollment of participants will be completed in 3 years. We expect to recruit 20 patients per month and to reach our target sample of 438 participants over a 21-month period. On average, this clinic initiates 40 patients >15 years of age on TB treatment per month. Pregnant women will be excluded from enrollment and will therefore not be exposed to additional risk of chest x-rays conducted for this study. Children under the age of 15 will not be enrolled in this study. Patients who are HIV positive will be enrolled in this study but will not be included in AIM 2 because of the potential interaction of antiretroviral medicines with TB treatment drugs. AIM 2 will include a subset of the total enrollment number.

During the study period, all patients initiating TB treatment who are identified through pre-screening to meet the study's eligibility criteria will be approached by study staff. If the patient is

interested, the study staff will describe the study to the patient and obtain written informed consent. After which the study staff will screen the patient for final eligibility and potential enrollment. Patients who consent to participate and are enrolled will be asked to complete a locator form that will enable us to aid with tracking participants for daily medication monitoring and subsequent assessments. Enrollment will occur ideally on the same day as TB treatment initiation. Patients who do not meet this study's clinical eligibility criteria but who are deemed to be capable and competent to provide informed consent will be asked about their interest in being part of other research studies conducted by the University of Cape Town's South African Tuberculosis Vaccine Initiative's (SATVI). If the patient expresses interest in participating in other studies and provides verbal consent for their contact details to be given to SATVI, they will be asked to provide their name and contact telephone number and this information will be passed on to SATVI. SATVI will then make further contact with the patient in order to assess whether they are potentially eligible to participate in other studies. We will not consent patients on behalf of SATVI but will merely refer them on (with the patient's verbal consent) for further assessment by the SATVI team for possible study eligibility.

A. AIM 1. All participants enrolled in this study will contribute to Aim 1. 438 participants will be enrolled over 3 years and followed up through standard, 6-month TB treatment and then every 3 months for 12 months after completion of treatment to evaluate post-treatment response and to perform behavioral assessments. Patients will get intensive follow-up weekly for the first 3 months of treatment to evaluate treatment response. We anticipate that 30% of eligible participants will be HIV positive based on a review of the clinic's electronic health record system. All participants will be assessed at baseline and throughout the study for alcohol use using both self-report and biologic measurements. We estimate that 30% of all participants will meet criteria for problem alcohol use. This will allow for evaluation of association between alcohol consumption and TB treatment outcomes.

B. AIM 2. A subset of the total enrolled participants, identified during screening and enrollment, will contribute to Aim 2. The first 200 participants who are eligible and agree to participate will be enrolled over 2 years. These participants will reflect the overall cohort of patients initiating TB treatment but will not include any HIV positive patients to remove potential drug interactions between TB drugs and antiretrovirals. These participants will contribute to information regarding TB drug PK. They will be asked to provide an 8-hour blood PK series one month into their TB treatment regimen

Inclusion of Women: Women will be included in this study. Pregnant women will be excluded from enrollment due to the additional risks associated with the diagnostic tests proposed (radiograph). We will do pregnancy testing on all women of reproductive age prior to enrollment. If a woman becomes pregnant after chest x-ray is performed and notifies study staff, this will be documented by the study but the woman will not be excluded from further participation.

Inclusion of Minorities: Participants in this study will be of all races and ethnic groups represented by TB patients in Worcester, South Africa.

Inclusion of Children: Children under the age of 15 will not be included in this study because drinking is not common under that age. The drinking age in South Africa is 18 but drinking practices are known to be common among adolescents under 18. Consent will be obtained from

a legal guardian for children under the age of 18 and separate consent will be obtained from children age 15-17 years of age. We will include ages 15-17 years because this age group includes individuals with alcohol problems and adult-type TB disease that would benefit from findings of this study. Parents/guardians and adolescents will be informed about the proposed project by receiving consent forms that describe what is involved by participating in the study, confidentiality protection procedures of the project, the purposes of the project, and the benefits and risks associated with participation. To minimise risk of harm for adolescent participants, adolescent information on alcohol use will not be shared with their parents/guardian. Each party will be informed of this project policy during the consent process.

5.2 Inclusion/Exclusion Criteria

To be eligible for this study, participants must meet all of the following inclusion criteria. Participants must be:

(1) at least 15 years old
(2) initiating TB treatment
(3) expect to remain in the local area for the next 2 years
(4) agree to comply with all study requirements, including provision of contact information and attendance at all study appointments
(5) provide written, informed consent to participate in the study if ≥18 years of age or written individual consent and separate parental consent if <18 years.
(6) has microbiologic confirmation (Xpert MTB/RIF, AFB smear, or mycobacterial culture) of MTB

Participants will be excluded from this study if:

(1) they have been treated for TB in the last 2 years (defined as cumulative treatment duration of one month or greater)
(2) they have RIF-resistant TB (RIF resistance will be known at screening from Xpert MTB/RIF or drug susceptibility testing [DST] from culture)
(3) they have extrapulmonary TB
(4) they have a contraindication to start on standard 4-drug therapy
(5) they are pregnant at study enrollment
(6) they do not have documented HIV status from the previous 6 months and refuse HIV testing
(7) they have a history of epilepsy
(8) they are HIV seropositive (for AIM 2 only)
(9) they do not speak English or Afrikaans

Participants will be excluded if they have been treated for TB in the last 2 years to exclude participants who may be relapsing from a previous episode of TB disease and also to avoid false positive Xpert MTB/RIF results. We want all participants to be receiving the same standard 6-month, 4 drug TB regimen, so both RIF-resistant TB and extrapulmonary TB cases are excluded. Patients with these types of TB will continue to receive the standard of care applicable and appropriate to their type of TB. Additionally, we will capture sputum time to positivity for pulmonary treatment response and do not have the means to track an extrapulmonary treatment response. We also exclude patients who have other contraindications

to start on standard therapy such as those with epilepsy. Pregnant women are excluded due to minimal risk of performing chest X-ray on them. We are excluding candidates who do not have documented HIV status from the previous 6 months and are unwilling to take an HIV test, as this is important information for Aim 2 of our study. Lastly, for Aim 2 only, we will exclude candidates if they are HIV seropositive to avoid potential drug interaction effects between ART and TB medications measured during PK studies.

6 STUDY PROCEDURES/EVALUATIONS

6.1 Study Procedures

Enrollment and initial assessment procedures: Immediately following study enrollment, participants will complete an interviewer-administered questionnaire, a sputum sample will be collected to obtain a mycobacterial isolate for AFB smear, MGIT growth, and MIC determination, a urine sample will be collected to assess recent drug use, and blood will be drawn to test for recent alcohol use using the phosphatidylethanol (PEth) test, and assess baseline health with CBC, HbA1c, AST/ALT, Cr, INR/PTT, and albumin. The results from the blood analysis baseline health assessment (except PEth results) will be provided to the TB Program. HbA1C will be measured as a baseline indicator of diabetes which has low detection rates in health clinics and which due to impact on the immune system, affect TB treatment outcomes and is therefore important to document. Participants who report substance use will be offered a referral to a local substance abuse treatment service (Toevlug Centre for Alcohol & Drug Dependence) and/or the social worker associated with the TB clinic. Patients who report or test positive for serious depression will also be referred to the social worker associated with the TB clinic. If not already done by the TB program, a chest radiograph will be obtained on each participant at Worcester Mediclinic, which will be saved in an electronic version together with interpretation. If already done by the TB program, the radiograph interpretation will be recorded. The enrollment process and initial biobehavioral assessment should take no longer than two hours, plus an additional thirty minutes for a chest radiograph. These activities will take place in a private room within the clinic. Following this assessment, participants will continue with their TB treatment. Study staff will also extract data on the participant's TB, HIV and physical health status, including known co-morbidities and active medications, from their clinic records.

Behavioral assessments.
Socio-demographic data will be collected on age, gender, race, residence, highest level of education completed, employment status, living situation, and current marital status.
Alcohol use: The Alcohol Timeline Follow Back (TLFB) technique will be used to assess recent drinking behavior.[52] The patient is asked to recall his/her daily alcohol consumption over a time period ranging from 7 days to 24 months prior to the interview. TLFB data can be used to assess frequency, quantity, volume, patterns and types of alcohol consumption over the specified time period. Studies have shown strong correlations between self-reported information on the TLFB and collateral reports and also excellent test-retest reliability for this measure.[53] We will also use the 12-item Alcohol Use Disorders Identification Test (AUDIT) to assess hazardous and harmful patterns of alcohol use.[54,55] We will inquire about age of initiation of alcohol use, and duration of regular and heavy alcohol consumption to assess the chronicity of alcohol use.
Tobacco use: The Fagerström Test for Nicotine Dependence (FTND), a 6-item scale, will be administered to assess current tobacco use and dependence.[56] Good correlations between FTND score and salivary cotinine have been found.[57]
Illicit drug use: We will inquire about lifetime use of any illicit drugs, types of illicit drugs used in the past month, age of initiation, number of days each illicit drug was used in the month and mode of use. In addition, the 11-item Drug Use Disorders Identification Test (DUDIT) will be administered to assess for harmful patterns of illicit drug use and dependence.[58,59]

Depression: The Center for Epidemiologic Studies Depression Scale (CES-D), a 20-item scale will be administered to measure depressive feelings and behaviors during the past week.[60]
Food security: The Household Hunger Scale, a 3-item instrument, will be used to assess food deprivation and security. Internal, external and cross-cultural validity of this scale has been demonstrated in South Africa.[61]
TB side effect screen: We will collect data on adverse reactions to first line drug regimens. Patients will be asked whether they have experienced any of the following since the last study visit: fevers, sweats, cough, rash, itching, phototoxicity, jaundice, nausea, vomiting, diarrhea, abdominal pain, loss of appetite, numbness/tingling of extremities, headache, dizziness, insomnia, joint pain, musculo-skeletal pain, or new infections. In addition, patients will be asked whether they have had other symptoms that are not listed above.

Mobile phone technology for monitoring of adherence to TB treatment. In South Africa, directly observed therapy, short course (DOTS) was adopted in 1995 as standard of care to ensure adherence to TB treatment; however the DOTS program has been inconsistently implemented and TB control remains poor.[62,63] In discussions with PI Jacobson, the Worcester CDC staff expressed concern about their DOTS program. They lack clinic staff to conduct DOTS and often rely on family members to observe treatment. They have noticed inconsistencies between medication counts (returned pills) and DOTS cards. They do not receive information on DOTS until patients return for their monthly clinic appointments, which is too late to put strategies in place to reduce non-adherence. To provide accurate and complete recording of adherence, we will employ our own DOTS workers to conduct treatment monitoring, and we will use mobile phone technology to permit centralized daily review of each participant's adherence to treatment. A GIS coordinate will be collected each time a DOTS worker logs a visit through the mobile phone app, allowing the study team to monitor their performance. If a participant is found to be adhering poorly, the TB Program will be informed.

Additional assessments. At the end of each of the 6 active treatment months, participants will complete an additional bio-behavioral assessment in a private room in the clinic. These assessments, which will be timed to coincide with the patient's regularly scheduled visit, will provide information about alcohol use since the last appointment and screen for TB treatment side effects. If there is a positive side effect screen at any assessment, we will check serum liver function (AST/ALT), creatinine, and CBC. If any results are outside a normal range or a patient has a significant new physical complaint, this information will be provided to the TB Program to be used at their discretion. In addition, we will extract data on weight and sputum smear results from participants' clinical records to assess for TB treatment response. During the active TB treatment period, if a participant reports that he/she had an unscheduled visit to a health care provider for a reason other than routine health care, or if the study team finds out otherwise, a study staff member will go to the clinic or hospital the participant visited to extract basic information on this unscheduled visit from the medical record. After the end of this treatment phase, participants will be contacted for interviews at 3, 6, 9, and 12 months post-treatment, during which they will be screened for any re-occurrence of TB symptoms and for alcohol use. At the 6 and 12 month post-treatment visits, the AUDIT, DUDIT, Fagerstrom Test, Household Hunger Scale, and CES-D will be reassessed. End of treatment will be determined by the clinic, after which the post-treatment period will commence. Participants with a positive TB symptom screen will be referred back to the clinic for microbiologic assessment, and we will extract the results of these assessments from their clinical records.

6.2 Laboratory Evaluations

6.2.1 Laboratory Evaluations/Assays

Determination of bacillary kill rates and MIC. Following standard procedures, spontaneous sputum specimens will be collected weekly from 1-12 weeks of treatment. Specimens will be transported once per week by a designated study member or accredited courier to co-I Warren's laboratory at the SAMRC. There they will be processed on liquid medium using BACTEC MGIT 960 system (Becton Dickinson, Sparks, MD). Time to positivity (TTP) will be recorded in days. These data will be used to build a time-to-event model to describe treatment response in these patients. On baseline, pre-treatment isolates, INH, RIF, and EMB MICs will be determined using the microdilution plate method. MIC for PZA will be determined using the BACTEC MGIT 960 PZA kit (Becton Dickinson Biosciences, Sparks) for patients co- enrolled in Aim 2; PZA DST will be performed on patients participating only in Aim 1. INH MICs will be completed while patients are still receiving therapy and any evidence of resistance will be transmitted to the clinical care team to decide if therapy will be adjusted based on their standard procedures. MICs and DST for the other 3 drugs will be batched and performed in years 1, 3, 4. All microbiologic data will be entered into a secure database from Warren's laboratory to ensure immediate transmission to BMC.

Biologic measurements. *Sputum AFB smear* will be recorded from clinical charts as well as verified in Warren's lab in standard fashion (negative, scanty, +, ++, +++) and *mycobacterial culture* from MGIT will be recorded as TTP (days). These measures will be used to estimate mycobacterial burden of TB disease at baseline and response to treatment. MICs for INH, RIF, EMB, and PZA will be performed to determine MTB drug susceptibility. An established scoring system will be used to quantify cavitary and non-cavitary disease burden from CXR at baseline as well.[64] Xpert MTB/RIF, AFB smear, and/or mycobacterial culture results will be recorded from the clinical chart documenting presence of TB and whether the *rpoB* gene is positive or negative for RIF resistance mutations or if there is evidence of RIF resistance via DST. Patients with RIF resistance will be excluded from this study but the number of patients with RIF resistance will be recorded to estimate incidence of resistance in this population. Biologic measures to capture end organ disease, including bone marrow suppression via white blood cell count, hemoglobin, platelets; liver function via ALT, AST, INR/PTT; renal function via Cr, as well as diabetes via HbA1c, will be recorded as continuous variables and dichotomized as normal or abnormal using standard cut-offs. Results will be shared with TB Program's clinical staff to be used at their discretion. BMI and albumin will be used with standard cut-offs to evaluate macro-nutritional status. Drug testing will be conducted at the baseline visit using urine dipstick tests and will provide indication of recent use of cannabis, methamphetamine, cocaine, opiates, amphetamines, and methaqualone (Mandrax). PEth testing will be conducted using dried blood spots (DBS) from venous blood. The testing will be performed at the United States Drug Testing Laboratories in Des Plaines, IL. The PEth tests will be done in batches throughout the study. PEth will be interpreted in a standard fashion using the 16:0/18.1 homologues.[65] Drug concentrations will be measured in McIlleron's laboratory by validated liquid chromatography-tandem mass spectrometry (LC-MS/MS) methods.

6.2.2 Special Assays or Procedures

PK phlebotomy and determination. After 4 weeks of therapy (but no more than 8 weeks, or as long as the participant is still in the intensive phase of treatment) participants enrolled for AIM 2 will be scheduled for an intensive phlebotomy day for PK sampling. Participants will be transported by research staff to Brewelskloof Hospital in Worcester. During that time the patient will have access to magazines, television, food, and water. One of the study nurses will manage and perform the procedures. Participants will be asked to arrive without eating and to not have taken their medication that morning; their TB drugs will be administered in person ideally under fasting conditions under observation. If a participant inadvertently eats or drinks the morning of PK, the nurse will ensure two hours elapse between time of last ingestion and when the first sample is taken and dose administered. The dosage times from the day before will be recorded from the study DOTS program. A breathalyzer test will be administered to record blood alcohol content that morning. Blood samples will be drawn by the study nurse immediately before the dose and then 1.5, 3, 5, and 8 hours after drug ingestion. Samples will be centrifuged and at least 1.2 ml of plasma will be stored in a -80° freezer on site within 30 minutes of collection. Samples will then be batch couriered on dry ice to the McIlleron's laboratory at UCT where plasma concentrations of INH, RIF, PZA, and EMB will be measured. Individual patient steady state 24-hour area under curve (AUC) and peak concentration (Cmax) will be identified from PK models implemented in NONMEM (Ellicott City, Maryland).

6.2.3 Specimen Shipping

All specimens will be coded and have no personal identifiers.

- Sputum specimens will be shipped to Rob Warren's laboratory at the SAMRC at Stellenbosch University, Tygerberg, South Africa

- Blood specimens for PK testing will be shipped to Helen McIlleron's laboratory at the University of Cape Town, Cape Town, South Africa

- Dried blood spots will be shipped to the United States Drug Testing Laboratories in Des Plaines, IL, USA for PEth testing

- Blood specimens for clinical tests (CBC, liver function tests, creatinine, albumin, HbA1C) will be shipped to and performed by a certified local clinical laboratory near Worcester, South Africa

7 STUDY SCHEDULE

7.1 Pre-Screening

We will review the medical records of patients after they have been evaluated for TB infection but before they return for potential TB treatment initiation in order to assess their eligibility for study inclusion based on clearly defined pre-screening criteria. To the degree possible, we will assess for the following criteria:

- Age
- Microbiologic confirmation of TB (e.g., positive Xpert MTB/RIF, AFB smear, mycobacterial culture)Location of TB disease (pulmonary vs extrapulmonary)
- History of treatment for active TB in the previous two years
- Pregnancy
- HIV status
- Epilepsy history

7.2 Screening

We will approach the potentially eligible participants identified through pre-screening during their Treatment Initiation visit with the TB program. During screening we will:

- Approach potential participants, provide information about the study, answer any potential questions
- Obtain and document consent for study participation.
- Review of medical records and brief assessment of medical history to confirm eligibility based on inclusion/exclusion criteria. This will include abstracting from their medical records HIV status, AFB smear, mycobacterial culture and Xpert MTB/RIF results, concomitant medications, and pregnancy status.
- Perform a urine pregnancy test (if patient is a woman of child-bearing potential)
- Eligible participants will complete locator form.

7.3 Enrollment/Baseline Visit (Treatment initiation visit)

- Interviewer-administered questionnaire to collect data about alcohol, tobacco, and illicit drug use, as well as participant demographics, depression, and food security
- Review of medical records to obtain medical history, HIV status, history of TB disease and treatment, physical health, and any other medical information considered relevant.
- Chest X-ray (if results are not available from the local TB program),
- Collection of a blood sample (total volume up to 20 mL).

- Collection of urine sample for drug testing.
- Collection of expectorated sputum sample for culture.

7.4 Weekly Visits (Treatment Weeks 1-12)

- Collection of expectorated sputum samples weekly to measure AFB smear status, time to positivity (TTP), and to assess rate of sterilization/culture and smear conversion. These will be collected by the DOTS workers at the patient home or other mutually decided location on weeks the patient is not attending clinic. On clinic visit weeks, these samples will be collected at the monthly clinic visit.

7.5 Daily Assessments (Months 1-6)

- DOTS worker will go to the patient's home or other mutually decided location to document TB drug ingestion 5 days/week (Monday to Friday) and obtain self-report information about weekend medication compliance

- DOTS worker will transmit this information (witnessed medication ingestion yes/no; if no, reason did not occur) via a mobile app to a secure server.

7.6 Single PK/PD Visit (In Treatment Week 4 – Treatment Week 8 window)

- 200 HIV-negative participants will participate in a single 8-hour TB drug PK testing
- Will be performed at Brewelskloof Hospital in Worcester; participants will be transported by study car if needed
- Five blood draws of up to 5 ml per draw during the 8 hour visit (baseline, 1.5, 3, 5, and 8 hours)

7.7 Monthly Follow-Up Visits (Treatment Month 1-6)

- Brief interviewer-administered questionnaire to collect data about alcohol use and TB drug side effects
- At month 6, interview-administered questionnaire to collect data about alcohol, tobacco, and illicit drug use, as well as participant demographics, depression, and food security
- Medical record review for participant's weight and AFB smear results
- At month 5, collection of expectorated sputum samples weekly to confirm AFB smear result documented by program
- Screen for TB drug side effects
- If positive side effect screen, collect a blood sample (up to 10 mL) to test for CBC, ALT, AST, and Cr and refer back to clinic for additional assessment

7.8 Post-Treatment Follow-Up (Months 3, 6, 9, 12 post treatment)

- Brief interviewer-administered questionnaire to collect data about alcohol use (months 3 and 9)
- Interviewer-administered questionnaire to collect data about alcohol, tobacco, and illicit drug use, as well as participant demographics, depression, and food security (months 6 and 12)
- TB symptom screen
- If positive TB symptom screen, participants will be referred back to clinic for TB assessment, and results of the assessment will be extracted from the medical record

7.9 Criteria for Discontinuation or Withdrawal of a Participant (or a Cohort), if applicable

- Clinical reasons believed to be life-threatening by the physician or the investigators
- Request by the participant to withdraw from study
- Participant judged by the investigator to be at significant risk of failing to comply with the provisions of the protocol as to cause harm to self or seriously interfere with the validity of the study results
- Participant moves and/or is transferred out to another clinic
- Participant shows bacteriologic evidence of treatment failure on month 5 sputum specimen
- Participant's treatment regimen duration is extended beyond the standard 6 months
- Participant is lost to follow-up for 2 months or longer
- Participant's TB disease relapses/recurs in the 12 month post-treatment follow-up period and is re-initiated on TB treatment
- At the discretion of the sponsor, IRB/REC or Office for Human Research Protections, investigators, or other government agencies as part of their duties to ensure that research participants are protected

8 ASSESSMENT OF OUTCOME MEASURES

8.1 Specification of the Appropriate Outcome Measures

8.1.1 Primary Outcome Measures

Aim 1: We will calculate the time to culture conversion, in patients with problem alcohol use compared to those without, at 12 weeks. This outcome has been shown to correlate well with poor treatment outcomes and to have detectable differences in alcohol-using populations.[7,66,67]

Aim 2: We will calculate the rate of decline in slowly dividing MTB subpopulations (β-slope or sterilizing activity) in the PD model. We will also investigate the effect of problem alcohol use on the PK measures Cmax and area under the curve (AUC) and the effect on sterilizing activity.

8.1.2 Secondary Outcome Measures

We will calculate the risk of poor final TB outcomes, defined as treatment failure, death, and relapse. We will report on barriers to adherence and individual patient level challenges based on daily reports from DOTS workers. We will determine whether any individuals who report alcohol abstinence have positive PEth biomarkers and re-classify these patients in the model. We will estimate the percentage of patients who develop side effects to the TB drugs and if there are significantly more side effects in patients who are acutely ingesting alcohol or who have a longer history of problem alcohol use relative to patients who do not drink.

9 SAFETY ASSESSMENT AND REPORTING

ICH E6 and South African GCP guidelines define an Adverse Event (AE) as any untoward medical event occurring in participant during his or her participation in the study irrespective of any causal relationship to any study procedure. An AE can therefore be any unfavorable and unintended sign (including abnormal laboratory finding), symptom or disease temporally associated with the administration of a study procedure (i.e., phlebotomy), whether or not related to the study procedure.

This study is largely observational, and is very low risk to participants. The only study procedures than can result in medical or psychological harm to participants are the phlebotomy, exposure to a single chest X-ray, and the potential risk for breach of confidentiality.

The occurrence of an AE may come to the attention of study personnel during study visits and interviews or by a recipient presenting for medical care. Any medical condition that is present at the time that the patient is screened should be considered as baseline and not reported as an AE.

9.1 Definition of Adverse Event (AE)

Adverse events will be monitored for each participant participating in the studies and attributed to the study intervention by the PIs according to the following categories:

a) Definite: Adverse event is clearly related to the intervention.
b) Probable: Adverse event is likely related to the intervention.
c) Possible: Adverse event may be related to the intervention.
d) Unlikely: Adverse event is likely not to be related to the intervention.
e) Unrelated: Adverse event is clearly not related to the intervention.

The following scale will be used in grading the severity of adverse events noted during the study:

1. Mild adverse event
2. Moderate adverse event
3. Severe adverse event

9.2 Definition of Serious Adverse Event (SAE)

In addition to grading the adverse event, the PI will determine whether the adverse event meets the criteria for a Serious Adverse Event (SAE). An adverse event is considered serious if it:

1. is life-threatening
2. results in in-patient hospitalization or prolongation of existing hospitalization
3. results in persistent or significant disability or incapacity

4. results in a congenital anomaly or birth defect
5. results in death
6. based upon appropriate medical judgment, may jeopardize the participant's health and may require medical or surgical intervention to prevent one of the other outcomes listed in this definition, or
7. adversely affects the risk/benefit ratio of the study

An adverse event may be graded as severe but still not meet the criteria for a Serious Adverse Event. Similarly, an adverse event may be graded as moderate but still meet the criteria for a Serious Adverse Event. The PI and mentorship team will determine the grade of the event as well as its "seriousness."

9.3 Reporting Procedures

In the unlikely event that such events occur, serious and unanticipated and related adverse events or unanticipated problems involving risks to participants or others will be reported in writing within 72 hours to the respective site IRB/REC, the DMID Medical Officer and the DMID Program Officer. The PI will apprise the investigative team and study personnel of all adverse events that occur during the conduct of this research project through regular study meetings and via email as they are reviewed by the principal investigator. The protocol's Program Officer and the appropriate IRB/RECs will be informed of moderate or greater in severity adverse events within three days of the event becoming known to the PI.

9.3.1 Serious Adverse Event Detection and Reporting

Serious adverse experiences (as defined in section 9.2 above) associated with the conduct of the study will be reported to the Ethics Committees in Boston and South Africa and the NIH (DMID Program Officer and DMID Medical Officer) in accordance with DHHS and international guidelines within three days of site awareness of their occurrence. Due to the laboratory/epidemiologic focus of the study, few serious adverse events related to study procedures are anticipated in this study.

9.3.2 Procedures to be Followed in the Event of Abnormal Laboratory Test Values or Abnormal Clinical Findings

We will report all laboratory results for routine blood tests (e.g., AST/ALT, Cr, albumin, complete blood count, HbA1c, INR/PTT) and INH MIC results to the TB treatment program in the clinic to interpret and use at their discretion. The clinic can then decide if it will take any steps to change clinical care based on those findings.

9.3.3 Type and Duration of the Follow-up of Participants After Adverse Events

AEs will be followed until resolution or stabilization even if this extends beyond the study-reporting period. Resolution of an AE is defined as the return to pretreatment status or stabilization of the condition with the expectation that it will remain chronic.

10 CLINICAL MONITORING STRUCTURE

10.1 Site Monitoring Plan

The study investigators are responsible for ensuring that, for this study:

- human participants' rights and well-being are protected;
- data are accurate, complete, and verifiable from source documents;
- the study complies with the protocol/amendment(s), sponsor requirements, ICH and South African Good Clinical Practice guidelines, and applicable regulatory requirements.

Site monitoring is conducted to ensure that the human subject protections, study and laboratory procedures, study intervention administration, and data collection processes are of high quality and meet sponsor, ICH and GCP guidelines and applicable regulations, and that the study is conducted in accordance with the protocol, protocol-specific MOP and applicable sponsor standard operating procedures. DMID, the sponsoring agency, or its designee will conduct site-monitoring visits as detailed in the clinical monitoring plan.

If the Sponsor deems necessary, monitoring visits by a sponsor-designated professional or monitor may occur at scheduled intervals prior to, during, and at study completion. Monitoring visits may include, but are not limited to, the review of regulatory files, CRFs, informed consent forms, medical and laboratory reports, and protocol compliance. Study monitors will meet with investigators to discuss any problems and actions to be taken and document visit findings and discussions. The investigational site will provide direct access to all study-related sites, source data/documents, and reports for the purpose of internal monitoring and in the event of auditing by the sponsor and inspection by regulatory authorities.

In addition, Boston Medical Center study staff may conduct auditing visits prior to, during, and at study completion.

11 STATISTICAL CONSIDERATIONS

11.1 Study Outcome Measures

Questions that will be addressed in Aim 1.

Primary questions: 1) We will estimate the prevalence of problem alcohol use among patients starting TB treatment and will evaluate changes in behavior during the course of and 1-yr post-treatment. 2) We will assess baseline TB disease characteristics associated with problem alcohol use, including TTP and CXR scores, and first line drug resistance prevalence. 3) We will calculate the time to culture conversion, in patients with problem alcohol use compared to those without, at 12 weeks. We will adjust for multiple potential confounders and mediators of these relationships as well, including tobacco and drug use, age, BMI/albumin, liver dysfunction, bone marrow suppression, HIV status, mycobacterial burden at baseline. We will stratify on adherence, defined as missing more or less than 20% of doses in the 12-week period.

Secondary questions: 4) We will calculate the risk of poor final TB outcomes, defined as treatment failure, death, and relapse. 5) We will explore whether there is a threshold effect on treatment outcomes for alcohol use and assess for the presence of any interactions with gender. 6) We will build models to assess whether acute alcohol ingestion, based on recent reported use, better predicts TB treatment outcomes compared to measures of chronic ingestion, including self-report measures and biologic indicators of end organ damage. 7) We will report on barriers to adherence and individual patient level challenges based on daily reports from DOTS workers. 8) We will determine whether any individuals who report alcohol abstinence have positive PEth biomarkers and re-classify these patients in the model. 9) We will estimate the percentage of patients who develop side effects to the TB drugs and if there are significantly more side effects in patients who are acutely ingesting alcohol or who have a longer history of problem alcohol use relative to patients who do not drink.

Questions to be addressed in Aim 2. 1) We will confirm preliminary findings of variability of specific drug PK among patients and the association with 12-week culture conversion and rate of decline in slowly dividing *M. tuberculosis* subpopulations (β-slope or sterilizing activity) in the PD model. 2) We will investigate the effect of problem alcohol use on the PK measures Cmax and area under the curve (AUC) and the effect on sterilizing activity, with adjustment for drug concentrations and mycobacterial drug MIC. 3) We will identify other potential modifiers of the alcohol-drug concentration-sterilization relationship, including BMI, gender, and age, and use these findings to build a prediction model of who is at highest risk of low PK with delayed PD. 4) We will also assess how these other modifiers explain variability in PK/PD seen in the control, non-problem alcohol population.

11.2 Sample Size Considerations

We powered the study using a logrank test to estimate the power to detect the difference in time to positive sputum culture with and without problematic alcohol consumption. **Fig. 2** shows the detectable hazard ratios (HR) with 291 participants (87 patients being problem alcohol drinkers), assuming a 5% drop-out rate at 12 weeks, and varying levels of survival among those without problematic alcohol consumption. In recent studies in this region, 20-30% of patients on standard therapy had mycobacterial growth in MGIT sputum cultures at 8 weeks of therapy (personal communication, Mark Hatherill, MD). If just 15% of those without problematic alcohol have a positive culture at 12 weeks, then we will be able to detect a HR of 0.63, indicating that 0.3 in the alcohol group had a positive culture at 12 weeks. For Aim 1, we will inflate the 291 by 5% to be able to stratify on patients with poor adherence (not fully default but take <80% of medications) and then inflate by an additional 30% for HIV positive patients. This would mean recruiting 438 patients for Aim 1 (307 HIV negative). For Aim 2, we will use the same model and recruit 200 participants from the 307 HIV negative, omitting any individuals who do not want the full day of phlebotomy or not able to participate due to their schedule. For this Aim, we will be able to detect a HR of 0.57, indicating 0.34 in the alcohol group having positive cultures at 12 weeks.

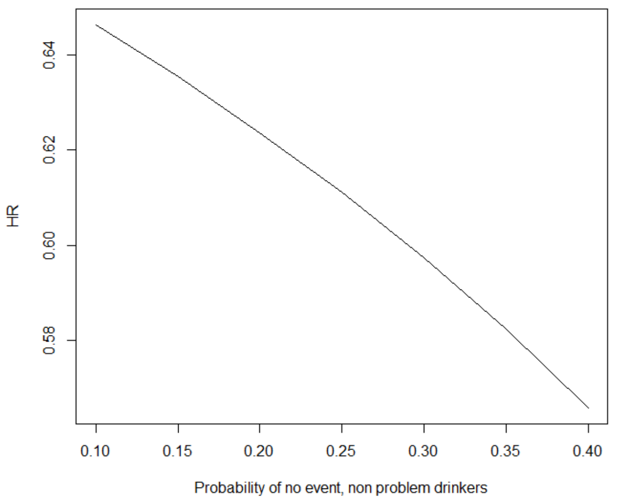

Figure 2. Detectable hazard ratio (HR) at 80% power for varying assumptions of positive culture probability

11.3 Participant Enrollment and Follow-Up

In our projections, we have factored in that 3-5% of patients will have RIF resistant TB and that a small proportion will refuse to participate. Based on findings from previous studies of heavy drinkers in this population, we anticipate no more than a 5% attrition rate at the end of the 12-week sputum sampling period. Myers and Parry have extensive experience in conducting longitudinal studies that involve tracking alcohol and other drug users.[68,69] We will follow procedures that have been used to successfully limit attrition, including a dedicated team to actively track participants taking their daily medications and between appointments, mobile phone, verbal, and face-to-face appointment reminders, and incentives to attend follow-up appointments. If a participant misses an appointment or 3 days of medication, study staff will use all of the contact information contained in the locator form to contact him/her.

11.4 Analysis Plan

Aim 1. We will first use basic descriptive statistics to estimate the prevalence of problem alcohol use in this population, as well as, baseline characteristics associated with problem alcohol use.

Consistent with our primary hypothesis, we will investigate the relationship between problem alcohol use and TB outcomes. Problem alcohol use will be assessed using both continuous and categorical measures from the follow-up visit data to assess the frequency of acute exposure and the regularity of alcohol ingestion. We will use basic bivariate tests to assess the association between alcohol use and the primary TB outcomes, as well as, the association between potential confounders and problem alcohol use. We will investigate the role of mediating factors by estimating the direct, indirect, and total effects of alcohol use on our outcomes of TTP and poor TB outcomes, using the classic approaches[70] as well as newer innovations.[70,71] We will control for HIV as a potential confounder and, if necessary, stratify our results by HIV status. We will model TTP using a poisson or negative binomial model, unless there is censoring, in which case we will use a Cox proportional hazards model. Poor TB outcomes will be modelled using logistic regression and Cox models, as appropriate. We will investigate potential threshold effects of alcohol by using a penalized spline for alcohol in the model and assessing nonlinear patterns graphically and with statistical tests.

We will further investigate the distinction between acute versus chronic effects of alcohol by fitting three different models: 1) model with acute consumption patterns as the exposure, 2) model with chronic measures of alcohol (i.e., liver function, years of drinking), and 3) model with both acute and chronic alcohol exposures, including an interaction between these types of exposures. We will use fit statistics (i.e. AIC and BIC) to assess the best fitting model and examine statistical significance of the interaction terms to detect potential synergy between the two classes of exposure. The outcome in these models will be time to culture conversion. We will also model the use of alcohol during the course of the study as a function of TB disease severity using a repeated measures model based on self-reported alcohol usage at each time point. All models will assess goodness-of-fit. Analyses will be performed in R (r-project.org) and SAS (SAS Institute, Cary, NC).

Aim 2. Our statistical modeler in Year 5 will build a time-to-event model to describe treatment response in patients as outlined in previous work by Co-Investigator McIlleron.59 In this model, the hazard of a positive culture result will be directly related to the bacillary load (measured using TTP data in sputum liquid cultures) predicted from the model. The nonlinear semi-mechanistic model will describe the biexponential decline in bacillary load using the α- and β-slopes. The β-slope is thought to reflect terminal sterilizing activity. Individual estimates of β-slope will be generated by the model for evaluation of the effects of drug exposure on sterilizing activity, in a multivariate adaptive regression splines (MARS) analysis. MARS is a non-parametric regression data analysis technique that combines recursive partitioning with fitting of splines to variables in the dataset. This method identifies the significant predictor variables, which enables splitting of the data into subregions of interest, while the spline fitting enables determination of relationships within the sub-regions. Drug interactions can be additive, synergistic or antagonistic. Potential predictors of the primary outcome (i.e. the β-slope) included in the MARS analysis will be problem alcohol use (versus non), AUC, Cmax, AUC/MIC, Cmax/MIC, individual drug MICs, the percentage of the 24-hour dosing interval that concentration exceeded MIC (%TMIC), gender, and BMI. Other variables found to significantly predict final outcomes in Aim 1 will also be considered for addition to this model.

12 ACCESS TO SOURCE DATA/DOCUMENTS

All study-related records (case report forms and laboratory results) will be maintained at the strictest level of confidentiality by all participating investigators, their staff, and the Sponsor and their agents. Participant identifiers will be replaced with a unique study identification number in all records to maintain confidentiality. All participant case report forms will be maintained in locked study-dedicated file cabinets at the study site at Ukwanda Rural Clinical School. Medical records will be maintained per standard practice in the hospital and participating medical clinics. Electronic records will be password protected and maintained on study-specific computers with limited access. Only encrypted files will be shared between BMC, SAMRC, and University of Cape Town.

The Sponsor (or Sponsor representative), the IRB/REC or Regulatory Agency representatives may consult and/or copy study documents in order to verify case report form data. By signing the informed consent document, the participant agrees to this process. Any documents to be copied will identify the participant only by unique study ID number, except in the case of the mobile DOTS app which will capture participant first name and first initial of surname. These study documents include, but are not limited to medical records (office, clinic or hospital) for the participants in this study. The clinical study site will permit access to such records.

13 QUALITY CONTROL AND QUALITY ASSURANCE

Following a written site quality management plan, the participating site is responsible for conducting routine quality assurance (QA) and quality control (QC) activities to internally monitor study progress and protocol compliance. The site principal investigator will provide direct access to all study-related sites, source data/data collection forms, and reports for the purpose of monitoring and auditing by the sponsor and/or its designee, and inspection by local and regulatory authorities. The site principal investigator will ensure all study personnel are appropriately trained and applicable documentations are maintained on site.

The investigators agree to be responsible for implementing and maintaining quality control and quality assurance systems with written SOPs to ensure that the proposed study is conducted and data are generated, documented, and reported in compliance with the protocol, accepted standards of Good Clinical Practice (GCP) and all applicable federal, state, and local laws and regulations relating to the conduct of this clinical study.

Following written SOPs, the investigators and staff will verify that the clinical study is conducted and data are generated, documented (recorded), and reported in compliance with the protocol, GCP, and the applicable regulatory requirements. The clinical sites at Worcester CDC, Brewelskloof Hospital, SAMRC, University of Cape Town, and BMC will provide direct access to all source data/documents and reports for the purpose of monitoring and auditing by the sponsor (or designee), and inspection by local and regulatory authorities.

The investigators will implement quality assurance (QA) and quality control (QC) procedures beginning with the data entry system and generate data quality control checks that will be run on the database. Any missing data or data anomalies will be communicated to the site(s) for clarification/resolution. QA/QC are essential elements in GCP guidelines regarding data management. These efforts run from data collection, laboratory conduct, and clinical conduct, to inventory maintenance and control. Database cleaning through automated query programs to identify potential problems (outliers, illogical errors, missing values, inconsistent responses, missing visit reports, etc.) with recorded data will be run by the data manager to generate query sheets. Queries will be transmitted to the clinical sites for response by the clinical staff who can check the original source documents for correction/verification. Identified corrections will be made to the original CRF, transmitted and verified by the data manager. Corrections will be made according to GCP.

14 ETHICS/PROTECTION OF HUMAN PARTICIPANTS

14.1 Ethical Considerations

Each Institution engaged in the research will hold a current Federal Wide Assurance (FWA) issued by the Office of Human Research Protections (OHRP) for human subjects research that is U.S. federally-funded.

14.2 Declaration of Helsinki

This study will be conducted in compliance with good clinical practice (GCP) as described in the International Conference on Harmonization (ICH) document "Guidance for Industry – E6 Good Clinical Practice: Consolidating Guidance," dated April 1996 and the South African guidelines for Good clinical practice. The study will also be carried out in keeping with local legal and regulatory requirements. All study procedures will comply with Declaration of Helsinki's Ethical Principles for Medical Research Involving Human Subjects.

14.3 Institutional Review Board

The protocol and informed consent forms that will be used must be approved by the ***Boston University Medical Campus Institutional Review Board*** (U.S. IRB), the ***SAMRC Ethics Committee,*** the ***University of Cape Town Health Research Ethics Committee***, and the ***Stellenbosch University Human Research Ethics Committee*** (South African RECs) before the study is initiated. The SAMRC, University of Cape Town, and Stellenbosch University's Health Research Ethics Committees are registered with the National Health Research Ethics Council (NHREC) in South Africa and with OHRP.

Other investigator responsibilities relative to the requirements of the IRB/REC include:

- Submit to the IRB/REC for review any advertisements that will be used to recruit patients.
- During the conduct of the study, submit progress reports to the IRB/REC, and request re-review of the study at least once a year.
- Report, in writing, to the IRB any serious adverse events (SAEs) that occur during the study.
- Inform the IRB/REC of any amendments in the protocol and obtain documented IRB/REC approval of those amendments.

14.4 Informed Consent Process

The investigator is responsible for the content of the informed consent form, but the content must be approved by the IRB/RECs (listed above) and the Sponsor. The content of the consent document must comply with the ICH and the South African Good Clinical Practices guidelines. It must also include any additional information required by local laws.

A designated study staff member is responsible for obtaining informed consent from each participant in the study. Consent/Assent forms will be translated into Afrikaans after IRB/REC approvals are obtained, after which a translator qualification form will be sent to the IRB/REC. Participants will be asked to read and review the Consent document. Upon reviewing the document, the staff member will explain the research study to the participant, describing in detail the study procedures and risks, and answer any questions that may arise. The participants will sign the informed consent document before any study-specific procedures are done. The participant may withdraw consent at any time throughout the course of the study. In case the participant is not able to read the consent form, the consent process will be performed in the presence of a literate witness unrelated to the study team. This witness will either be a family member or friend accompanying the patient or another person from the clinic who will be impartial and unrelated to the study. The witness will be asked to sign the consent form to verify that the consent process was properly performed and that the consent seemed to have been understood and given by free will. A copy of the informed consent document will be given to participants for their records. The rights and welfare of the participants will be protected by emphasizing to them that the quality of their medical care will not be adversely affected if they decline to participate in this study.

14.4.1 Informed Consent Process

Parental consent will be obtained for children <18 years of age enrolled in this study. Separate consent will be obtained from minor children aged 15 to 17 years. Minors will be asked to re-consent when they turn 18 years old.

14.5 Exclusion of Women, Minorities, and Children (Special Populations)

Special care will be taking with these populations and the consent forms will capture required elements according to 45 CFR 46 Subparts B and D.

Pregnant women will be excluded from enrollment due to the additional risks associated with the diagnostic tests proposed (radiograph). We will perform a pregnancy test before the radiograph in all women of child bearing age. If a woman becomes pregnant after enrollment she will be not be removed from this study.

Participants in this study will be of all races and ethnic groups represented by tuberculosis patients in Worcester, South Africa.

Children under the age of 15 will not be included in this study because drinking is not common under that age.

14.6 Participant Confidentiality

Participant records will be maintained at the strictest level of confidentiality by all participating investigators, their staff, and the Sponsor and their agents. Participant identifiers will be replaced with a unique study identification number in all records to maintain confidentiality. Participant identifiers collected in a pre-screening log will be blacked out immediately after a patient is deemed to be potentially eligible and approached for screening, or deemed to be ineligible. All participant case report forms will be maintained in locked study-dedicated file cabinets at the Ukwanda site. Medical records will be maintained per standard practice in the medical clinic (Worcester CDC). Participant consent will include access to medical records maintained at these facilities. Electronic records will be password protected and maintained on study-specific computers with limited access. Participant records, not limited to medical records (office, clinic or hospital), will not be available for health insurance companies, employers, or to other individuals.

The Sponsor (or Sponsor representative), the IRB/REC or Regulatory Agency representatives may consult and/or copy study documents in order to verify case report form data. By signing the informed consent document, the participant agrees to this process. Any documents including medical records (office, clinic or hospital) to be copied will identify the participant only by unique study ID number. The clinical study site will permit access to such records.

All biological samples will be stored in South Africa under the supervision of the site PI. Samples will be stored in -80ºC freezers or liquid nitrogen for a period of five (5) years post-ending the project. To assure patient confidentiality, stored samples will be labeled using the study identification number. Patients will be encouraged to maintain their address/contact updated in order to allow future contact to inform them about relevant research information or to obtain a new specific informed consent for stored sample use in a new project.

14.7 Future Use of Stored Specimens

We do not plan to keep samples past the five years post-project ending.

15 DATA HANDLING AND RECORD KEEPING

It is anticipated that participants will have been recruited and completed the study in approximately 18 months from initial screening to final visit. Data will be collected, maintained, and analyzed by investigators at SAMRC, University of Cape Town, Stellenbosch University, BU, and BMC. Files will be kept in dedicated locked file cabinets. Medical records will be stored at the clinical sites per standard operating practices. Source CRFs will be maintained at SAMRC.

Electronic data and files will be maintained on password protected computers. Archival protection of all electronic files (CRFs and electronic databases) will be maintained on servers at Boston University that meet HIPAA compliance related to protected health information (PHI).

An automated audit trail will be maintained by REDCap. REDCap will automatically log and document all user activity (e.g., entering data, exporting data, modifying a field, running a report, or add/modifying a user) as well as queries and corrections made to every data point. If errors are found, corrections will be made according to GCP standards both to the original CRF as well as to the electronic database.

15.1 Data Management Responsibilities

It is the responsibility of the clinical sites and study coordinator to enter completed CRFs into the REDCap database within five to seven business days of completion. Timely submission allows for prompt query generation and resolution and securing of data. Investigators and staff will manage data capture, management, query submission/resolution, quality control and assurance, and provide statistical analysis of all results.

15.2 Data Capture Methods

All study forms will be linked to each other by a unique participant number. The pen-and-paper forms will be stored in locked filing cabinets at the Ukwanda study site, with consent and locator forms being stored separately from questionnaires and biological screening forms. All forms will be transported from the clinical site at Worcester CDC back to the Ukwanda study site and secured in locked filing cabinets at the end of each working day. Quality control methods will be implemented consistently over time to ensure high quality of the incoming data stream and to avoid drift in data reporting/ascertainment practices. We will use REDCap (Research Electronic Data Capture) for data entry and database management, hosted on the Boston University Medical Center (BUMC) and maintained by study staff at Boston Medical Center

The MRC site is experienced in conducting clinical studies to international standards (R01 HD058320; R21DA03054; 1R01DA032061-04-S1). UCT and the Warren laboratory at Stellenbosch University will transmit results following the same procedures. Any queries on data will be transmitted back on a weekly basis via REDCap; the project manager will then work to verify any discrepant data at the clinical site. All laboratory data queries will be submitted to McIlleron or Warren to review with their technician to assure validity.

15.3 Types of Data

Data collected for this study will include:

1. Clinical questionnaire and evaluations*;
2. Behavioral assessments;
3. Observational Adherence data;
4. Sputum from TB cases for AFB smear, culture, and MIC testing*;
5. Chest X-ray from TB cases*;
6. HIV serology from TB cases*;
7. Serum for biologic measures;
8. Serum for PK testing;
9. Dried blood spot for PEth testing
10. Urine for pregnancy and drug testing

(*Procedures conducted all or in part by the National TB Program)

15.4 Timing/Reports

Reports on the enrollment and progress of study activities will be sent to NIAID as requested.

15.5 Study Records Retention

The investigator will make study data accessible to the Sponsor or its representatives, and to other Regulatory agency inspectors. All study-related records (patient charts, case report forms, and other study records) will be retained until disposal is authorized by NIAID and/or DBT.

15.6 Protocol Deviations

A protocol violation/deviation is any nonadherence to the clinical trial protocol, GCP, or Manual of Procedures requirements. Violations are deviations that threaten the integrity of the study, while deviations are those which do not. As a result of violations or deviations, corrective actions will be developed by the site and implemented promptly. Protocol violations should be reported within 7 days to the local IRB/REC per the local IRB/REC guidelines. Any protocol deviation that meets reporting requirements of the local IRB/REC will also be reported with the same timeliness to the Boston Medical Center IRB and to the DMID Program Officer. All deviations from the Protocol must be addressed in the study participant source documents. The documentation should include the reason(s) for the deviation and all attempts to prevent or correct the deviation.

16 PUBLICATION POLICY

Following completion of the study, the investigators plan to publish the results of this research in peer-reviewed scientific journals. Investigational plans, protocols, and data related to this study will be treated as confidential information. Authorship will be extended to the following individuals: study investigators, site PIs, and other individuals having major contribution to the study design, implementation, data analysis, and preparation of the written manuscript.

17 LITERATURE REFERENCES

1. World Health Organization. Global Tuberculosis Report 2013 [Internet]. Geneva; 2013. Available from: http://apps.who.int/iris/bitstream/10665/91355/1/9789241564656_eng.pdf?ua=1

2. Health Systems Trust [Internet]. [cited 2014 Oct 1]. Available from: http://www.hst.org.za/

3. Statistics South Africa. Mortality and causes of death in South Africa, 2010: Findings from death notification [Internet]. Pretoria; 2013. Available from: http://www.statssa.gov.za/publications/P03093/P030932010.pdf

4. Wood R, Lawn SD, Caldwell J, Kaplan R, Middelkoop K, Bekker L-G. Burden of new and recurrent tuberculosis in a major South African city stratified by age and HIV-status. PloS One. 2011;6(10):e25098. PMCID: PMC3189963

5. Creswell J, Raviglione M, Ottmani S, Migliori GB, Uplekar M, Blanc L, et al. Tuberculosis and noncommunicable diseases: neglected links and missed opportunities. Eur Respir J. 2011 May;37(5):1269–1282. PMID: 20947679

6. Parry C, Rehm J, Poznyak V, Room R. Alcohol and infectious diseases: an overlooked causal linkage? Addiction 2009; 104: 331–2.

7. Volkmann T, Moonan PK, Miramontes R, Oeltmann JE. Tuberculosis and excess alcohol use in the United States, 1997-2012. Int J Tuberc LUNG Dis. 2015 Jan;19(1):111–119. PMID: 25519800

8. WHO | Global status report on alcohol and health 2014 [Internet]. WHO. [cited 2014 Sep 30]. Available from: http://www.who.int/substance_abuse/publications/global_alcohol_report/en/

9. Rehm J, Samokhvalov AV, Neuman MG, Room R, Parry C, Lönnroth K, et al. The association between alcohol use, alcohol use disorders and tuberculosis (TB). A systematic review. BMC Public Health. 2009;9:450. PMCID: PMC2796667

10. Stein DJ, Seedat S, Herman A, Moomal H, Heeringa SG, Kessler RC, et al. Lifetime prevalence of psychiatric disorders in South Africa. Br J Psychiatry. 2008 Feb 1;192(2):112–117. PMCID: PMC2718689

11. Gossage JP, Snell CL, Parry CDH, Marais A-S, Barnard R, de Vries M, et al. Alcohol use, working conditions, job benefits, and the legacy of the “Dop” system among farm workers in the Western Cape Province, South Africa: hope despite high levels of risky drinking. Int J Environ Res Public Health. 2014 Jul;11(7):7406–7424. PMCID: PMC4113884

12. McLoughlin J-A, Little F, Mazok C, Parry C, London L. Prevalence of and associations with papsak wine consumption among farm workers in the Western Cape Province, South Africa. J Stud Alcohol Drugs. 2013 Nov;74(6):879–888. PMID: 24172114

13. London L. Alcohol consumption amongst South African farm workers: a challenge for post-apartheid health sector transformation. Drug Alcohol Depend. 2000 May 1;59(2):199–206. PMID: 10891634

14. Friedman LN, Sullivan GM, Bevilaqua RP, Loscos R. Tuberculosis screening in alcoholics and drug addicts. Am Rev Respir Dis. 1987 Nov;136(5):1188–1192. PMID: 3674581

15. Lönnroth K, Williams BG, Stadlin S, Jaramillo E, Dye C. Alcohol use as a risk factor for tuberculosis - a systematic review. BMC Public Health. 2008;8:289. PMCID: PMC2533327

16. De Albuquerque M, Ximenes RAD, Lucena-Silva N, de Souza WV, Dantas AT, Dantas OMS, et al. Factors associated with treatment failure, dropout, and death in a cohort of tuberculosis patients in Recife, Pernambuco State, Brazil. Cad Saude Publica. 2007;23(7):1573–1582. PMID: 17572806

17. Duraisamy K, Mrithyunjayan S, Ghosh S, Nair SA, Balakrishnan S, Subramoniapillai J, et al. Does Alcohol Consumption during Multidrug-Resistant Tuberculosis Treatment Affect Outcome? A Population-based Study in Kerala, India. Ann Am Thorac Soc [Internet]. 2014 Apr; Available from: http://www.ncbi.nlm.nih.gov/pubmed/24735096 PMID: 24735096

18. Kendall EA, Theron D, Franke MF, van Helden P, Victor TC, Murray MB, et al. Alcohol, hospital discharge, and socioeconomic risk factors for default from multidrug resistant tuberculosis treatment in rural South Africa: a retrospective cohort study. PloS One. 2013;8(12):e83480. PMCID: PMC3862731

19. Przybylski G, Dąbrowska A, Trzcińska H. Alcoholism and other socio-demographic risk factors for adverse TB-drug reactions and unsuccessful tuberculosis treatment - data from ten years' observation at the Regional Centre of Pulmonology, Bydgoszcz, Poland. Med Sci Monit Int Med J Exp Clin Res. 2014 Jan;20:444–53. PMCID: PMC3965286

20. Shin SS, Pasechnikov AD, Gelmanova IY, Peremitin GG, Strelis AK, Mishustin S, et al. Treatment outcomes in an integrated civilian and prison MDR-TB treatment program in Russia. Int J Tuberc Lung Dis Off J Int Union Tuberc Lung Dis. 2006 Apr;10(4):402–408. PMID: 16602404

21. Fleming MF, Krupitsky E, Tsoy M, Zvartau E, Brazhenko N, Jakubowiak W, et al. Alcohol and drug use disorders, HIV status and drug resistance in a sample of Russian TB patients. Int J Tuberc Lung Dis Off J Int Union Tuberc Lung Dis. 2006 May;10(5):565–570. PMCID: PMC1570181

22. Mathew TA, Ovsyanikova TN, Shin SS, Gelmanova I, Balbuena DA, Atwood S, et al. Causes of death during tuberculosis treatment in Tomsk Oblast, Russia. Int J Tuberc Lung Dis. 2006 Aug 1;10(8):857–863. PMID: 16898369

23. Kattan JA, Sosa LE, Lobato MN. Tuberculosis mortality: death from a curable disease, Connecticut, 2007-2009. Int J Tuberc Lung Dis Off J Int Union Tuberc Lung Dis. 2012 Dec;16(12):1657–1662. PMID: 23131265

24. Jakubowiak WM, Bogorodskaya EM, Borisov SE, Borisov ES, Danilova ID, Danilova DI, et al. Risk factors associated with default among new pulmonary TB patients and social support in six Russian regions. Int J Tuberc Lung Dis Off J Int Union Tuberc Lung Dis. 2007 Jan;11(1):46–53. PMID: 17217129

25. Ribeiro Macedo L, Reis-Santos B, Riley LW, Maciel EL. Treatment outcomes of tuberculosis patients in Brazilian prisons: a polytomous regression analysis. Int J Tuberc Lung Dis Off J Int Union Tuberc Lung Dis. 2013 Nov;17(11):1427–1434. PMID: 24125446

26. Santha T, Garg R, Frieden TR, Chandrasekaran V, Subramani R, Gopi PG, et al. Risk factors associated with default, failure and death among tuberculosis patients treated in a DOTS programme in Tiruvallur District, South India, 2000. Int J Tuberc Lung Dis Off J Int Union Tuberc Lung Dis. 2002 Sep;6(9):780–788. PMID: 12234133

27. Kliiman K, Altraja A. Predictors and mortality associated with treatment default in pulmonary tuberculosis. Int J Tuberc Lung Dis. 2010 Apr 1;14(4):454–463. PMID: 20202304

28. Srivastava S, Pasipanodya JG, Meek C, Leff R, Gumbo T. Multidrug-resistant tuberculosis not due to noncompliance but to between-patient pharmacokinetic variability. J Infect Dis. 2011 Dec 15;204(12):1951–1959. PMCID: PMC3209814

29. Pasipanodya JG, Srivastava S, Gumbo T. Meta-Analysis of Clinical Studies Supports the Pharmacokinetic Variability Hypothesis for Acquired Drug Resistance and Failure of Antituberculosis Therapy. Clin Infect Dis Off Publ Infect Dis Soc Am. 2012 Jul 15;55(2):169–177. PMCID: PMC3491771

30. Gumbo T, Dona CSWS, Meek C, Leff R. Pharmacokinetics-pharmacodynamics of pyrazinamide in a novel in vitro model of tuberculosis for sterilizing effect: a paradigm for faster assessment of new antituberculosis drugs. Antimicrob Agents Chemother. 2009 Aug;53(8):3197–3204. PMCID: PMC2715614

31. Gumbo T, Louie A, Deziel MR, Liu W, Parsons LM, Salfinger M, et al. Concentration-dependent Mycobacterium tuberculosis killing and prevention of resistance by rifampin. Antimicrob Agents Chemother. 2007 Nov;51(11):3781–3788. PMCID: PMC2151424

32. Shandil RK, Jayaram R, Kaur P, Gaonkar S, Suresh BL, Mahesh BN, et al. Moxifloxacin, ofloxacin, sparfloxacin, and ciprofloxacin against Mycobacterium tuberculosis: evaluation

of in vitro and pharmacodynamic indices that best predict in vivo efficacy. Antimicrob Agents Chemother. 2007 Feb;51(2):576–582. PMCID: PMC1797767

33. Chideya S, Winston CA, Peloquin CA, Bradford WZ, Hopewell PC, Wells CD, et al. Isoniazid, rifampin, ethambutol, and pyrazinamide pharmacokinetics and treatment outcomes among a predominantly HIV-infected cohort of adults with tuberculosis from Botswana. Clin Infect Dis Off Publ Infect Dis Soc Am. 2009 Jun 15;48(12):1685–1694. PMCID: PMC3762461

34. Weiner M, Benator D, Burman W, Peloquin CA, Khan A, Vernon A, et al. Association between acquired rifamycin resistance and the pharmacokinetics of rifabutin and isoniazid among patients with HIV and tuberculosis. Clin Infect Dis Off Publ Infect Dis Soc Am. 2005 May 15;40(10):1481–1491. PMID: 15844071

35. Pasipanodya JG, McIlleron H, Burger A, Wash PA, Smith P, Gumbo T. Serum drug concentrations predictive of pulmonary tuberculosis outcomes. J Infect Dis. 2013 Nov 1;208(9):1464–1473. PMCID: PMC3789573

36. Koriakin VA, Sokolova GB, Grinchar NA, Iurchenko LN. [Pharmacokinetics of isoniazid in patients with pulmonary tuberculosis and alcoholism]. Probl Tuberk. 1986;(12):43–46. PMID: 3823081

37. Olsen H, Mørland J. Ethanol-induced increase in drug acetylation in man and isolated rat liver cells. Br Med J. 1978 Nov 4;2(6147):1260–1262. PMCID: PMC1608489

38. Massi MN, Wahyuni S, Halik H, Anita, Yusuf I, Leong FJ, et al. Drug resistance among tuberculosis patients attending diagnostic and treatment centres in Makassar, Indonesia. Int J Tuberc Lung Dis Off J Int Union Tuberc Lung Dis. 2011 Apr;15(4):489–495. PMID: 21396208

39. Happel KI, Nelson S. Alcohol, immunosuppression, and the lung. Proc Am Thorac Soc. 2005;2(5):428–432. PMID: 16322595

40. Dannenberg AM. Immune mechanisms in the pathogenesis of pulmonary tuberculosis. Rev Infect Dis. 1989 Apr;11 Suppl 2:S369–378. PMID: 2496453

41. Bermudez LE, Young LS. Ethanol augments intracellular survival of Mycobacterium avium complex and impairs macrophage responses to cytokines. J Infect Dis. 1991 Jun;163(6):1286–1292. PMID: 2037794

42. Bermudez LE. Effect of ethanol on the interaction between the macrophage and Mycobacterium avium. Alcohol Fayettev N. 1994 Apr;11(2):69–73. PMID: 8204205

43. Lieber CS. The influence of alcohol on nutritional status. Nutr Rev. 1988 Jul;46(7):241–254. PMID: 3045703

44. Peltzer K, Louw J, McHunu G, Naidoo P, Matseke G, Tutshana B. Hazardous and harmful alcohol use and associated factors in tuberculosis public primary care patients in

South Africa. Int J Environ Res Public Health. 2012 Sep;9(9):3245–3257. PMCID: PMC3499864

45. Crews FT, Bechara R, Brown LA, Guidot DM, Mandrekar P, Oak S, et al. Cytokines and Alcohol. Alcohol Clin Exp Res. 2006 Apr 1;30(4):720–730. PMID: 16573591

46. Mason CM, Dobard E, Zhang P, Nelson S. Alcohol exacerbates murine pulmonary tuberculosis. Infect Immun. 2004 May;72(5):2556–2563. PMCID: PMC387844

47. Chigutsa E, Patel K, Denti P, Visser M, Maartens G, Kirkpatrick CMJ, et al. A time-to-event pharmacodynamic model describing treatment response in patients with pulmonary tuberculosis using days to positivity in automated liquid mycobacterial culture. Antimicrob Agents Chemother. 2013 Feb;57(2):789–795. PMCID: PMC3553722

48. McIlleron H, Wash P, Burger A, Norman J, Folb PI, Smith P. Determinants of rifampin, isoniazid, pyrazinamide, and ethambutol pharmacokinetics in a cohort of tuberculosis patients. Antimicrob Agents Chemother. 2006 Apr;50(4):1170–1177. PMCID: PMC1426981

49. Jeremiah K, Denti P, Chigutsa E, Faurholt-Jepsen D, PrayGod G, Range N, et al. Nutritional supplementation increases rifampin exposure among tuberculosis patients coinfected with HIV. Antimicrob Agents Chemother. 2014 Jun;58(6):3468–3474. PMCID: PMC4068463

50. Parry CDH, Gossage JP, Marais A-S, Barnard R, de Vries M, Blankenship J, et al. Comparison of baseline drinking practices, knowledge, and attitudes of adults residing in communities taking part in the FAS prevention study in South Africa. Afr J Drug Alcohol Stud. 2012 Jul;11(2):65–76. PMCID: PMC4225715

51. Kader R, Seedat S, Koch JR, Parry CD. A preliminary investigation of the AUDIT and DUDIT in comparison to biomarkers for alcohol and drug use among HIV-infected clinic attendees in Cape Town, South Africa. Afr J Psychiatry. 2012 Sep;15(5):346–351. PMID: 23044889

52. Sobell LC, Sobell MB. Timeline Follow-Back. In: Litten RZ, Allen JP, editors. Meas Alcohol Consum [Internet]. Humana Press; 1992 [cited 2014 Oct 1]. p. 41–72. Available from: http://link.springer.com/chapter/10.1007/978-1-4612-0357-5_3

53. Sobell LC, Sobell MB, Leo GI, Cancilla A. Reliability of a timeline method: assessing normal drinkers' reports of recent drinking and a comparative evaluation across several populations. Br J Addict. 1988 Apr;83(4):393–402. PMID: 3395719

54. Babor T, Higgins-Biddle J, Saunders J, Monteiro M. The Alcohol Use Disorders Identification Test, Guidelines for Use in Primary Care [Internet]. World Health Organization: Department of Mental Health and Substance Dependence; 2001 [cited 2014 Oct 1]. Available from: http://www.talkingalcohol.com/files/pdfs/WHO_audit.pdf

55. Pengpid S, Peltzer K, van der Heever H, Skaal L. Screening and brief interventions for hazardous and harmful alcohol use among university students in South Africa: results from a randomized controlled trial. Int J Environ Res Public Health. 2013 May;10(5):2043–2057. PMCID: PMC3709363

56. Heatherton TF, Kozlowski LT, Frecker RC, Fagerström KO. The Fagerström Test for Nicotine Dependence: a revision of the Fagerström Tolerance Questionnaire. Br J Addict. 1991 Sep;86(9):1119–1127. PMID: 1932883

57. Meneses-Gaya IC de, Zuardi AW, Loureiro SR, Crippa JA de S. Psychometric properties of the Fagerström Test for Nicotine Dependence. J Bras Pneumol Publicaçáo Of Soc Bras Pneumol E Tisilogia. 2009 Jan;35(1):73–82. PMID: 19219334

58. Berman AH, Bergman H, Palmstierna T, Schlyter F. Evaluation of the Drug Use Disorders Identification Test (DUDIT) in criminal justice and detoxification settings and in a Swedish population sample. Eur Addict Res. 2005;11(1):22–31. PMID: 15608468

59. Voluse AC, Gioia CJ, Sobell LC, Dum M, Sobell MB, Simco ER. Psychometric properties of the Drug Use Disorders Identification Test (DUDIT) with substance abusers in outpatient and residential treatment. Addict Behav. 2012 Jan;37(1):36–41. PMID: 21937169

60. Radloff LS. The CES-D Scale A Self-Report Depression Scale for Research in the General Population. Appl Psychol Meas. 1977 Jun 1;1(3):385–401.

61. Deitchler M, Ballard T, Swindale A, Coates J. Introducing a Simple Measure of Household Hunger for Cross-Cultural Use. 2011 Feb; Available from: http://www.fao.org/fileadmin/user_upload/wa_workshop/docs/HH_Hunger_Scale.pdf

62. Abdool Karim SS, Churchyard GJ, Karim QA, Lawn SD. HIV infection and tuberculosis in South Africa: an urgent need to escalate the public health response. Lancet. 2009 Sep 12;374(9693):921–933. PMCID: PMC2803032

63. Ntshanga SP, Rustomjee R, Mabaso MLH. Evaluation of directly observed therapy for tuberculosis in KwaZulu-Natal, South Africa. Trans R Soc Trop Med Hyg. 2009 Jun;103(6):571–574. PMID: 19394664

64. Ralph AP, Ardian M, Wiguna A, Maguire GP, Becker NG, Drogumuller G, et al. A simple, valid, numerical score for grading chest x-ray severity in adult smear-positive pulmonary tuberculosis. Thorax. 2010 Oct;65(10):863–869. PMID: 20861290

65. Hahn JA, Dobkin LM, Mayanja B, Emenyonu NI, Kigozi IM, Shiboski S, et al. Phosphatidylethanol (PEth) as a biomarker of alcohol consumption in HIV positives in sub-Saharan Africa. Alcohol Clin Exp Res. 2012 May;36(5):854–862. PMCID: PMC3310261

66. Daley P, Jagannathan V, John KR, Sarojini J, Latha A, Vieth R, et al. Adjunctive vitamin D for treatment of active tuberculosis in India: a randomised, double-blind, placebo-controlled trial. Lancet Infect Dis. 2015 May;15(5):528–534. PMID: 25863562

67. Epstein MD, Schluger NW, Davidow AL, Bonk S, Rom WN, Hanna B. Time to detection of Mycobacterium tuberculosis in sputum culture correlates with outcome in patients receiving treatment for pulmonary tuberculosis. Chest. 1998 Feb;113(2):379–386. PMID: 9498955

68. Wechsberg WM, Jewkes R, Novak SP, Kline T, Myers B, Browne FA, et al. A brief intervention for drug use, sexual risk behaviours and violence prevention with vulnerable women in South Africa: a randomised trial of the Women's Health CoOp. BMJ Open. 2013;3(5). PMCID: PMC3657672

69. Wechsberg WM, Luseno WK, Karg RS, Young S, Rodman N, Myers B, et al. Alcohol, cannabis, and methamphetamine use and other risk behaviours among Black and Coloured South African women: a small randomized trial in the Western Cape. Int J Drug Policy. 2008 Apr;19(2):130–139. PMCID: PMC2435299

70. Baron RM, Kenny DA. The moderator-mediator variable distinction in social psychological research: conceptual, strategic, and statistical considerations. J Pers Soc Psychol. 1986 Dec;51(6):1173–1182. PMID: 3806354

71. VanderWeele TJ. A unification of mediation and interaction: a 4-way decomposition. Epidemiol Camb Mass. 2014 Sep;25(5):749–761. PMCID: PMC422027

72. Valeri L, Vanderweele TJ. Mediation analysis allowing for exposure-mediator interactions and causal interpretation: theoretical assumptions and implementation with SAS and SPSS macros. Psychol Methods. 2013 Jun;18(2):137–150. PMCID: PMC3659198

Appendix A: Study Schedule

Procedures	Pre-screening	Screening	Baseline	Follow-Up Schedule: Daily during treatment	Weekly during treatment	Monthly during treatment	Aim 2 PK visit	Post treatment
Assessment of Eligibility Criteria	X	X						
Signed Consent Form		X						
Pregnancy test		X						
Complete locator form		X						
Participant interview		X	X			X		
Medical records review		X	X			X		
Biobehavioral screen			X			X		X
Chest radiograph			X					
Drug side effect screen						X		
Sputum collection			X		X[2]	X[3]		
Blood collection			X			X[4]	X	
Witnessed pill ingestion + count				X				
Symptom screen and recent alcohol use questions[5]								X

[2]Weekly sputum collected only Weeks 1-12

[3]Sputum will be collected at the month five study visit to confirm the results of the routine smear done at month five of treatment by the national TB program

[4]Blood collection will only occur if participant has TB drug side effect symptoms. After study visit, participant will be referred to TB program for additional assessment.

[5]Screening for TB symptoms and assessment of recent (previous 2 weeks) alcohol consumption will be done every three months after end of treatment for a total of 12 months (post treatment months 3, 6, 9, and 12)
