# Supplementary material for: Impact of alcohol consumption on tuberculosis treatment outcomes: a prospective longitudinal cohort study protocol
Source: BMC Infect Dis. 2018 Sep 29;18:488. doi: 10.1186/s12879-018-3396-y (PMC6162918; doi:10.1186/s12879-018-3396-y)
Supplement: Supplementary file 2 — Ethical approval reference numbers. This document contains a list of the specific names and reference numbers for all ethical bodies that approved the study in the various participating and involved centers. (DOCX 13 kb) [file 12879_2018_3396_MOESM2_ESM.docx]

IRB Institutions that approved the TRUST protocol

| **IRB Institution** | **FWA number** | **Registration number** |
| --- | --- | --- |
| **Boston University Medical Campus** | **00000301** | IRB00000377 |
| **University of Capetown**  **Faculty of Health Science**  **Human Research Ethics Committee** | 00001637 | IRB00001938 |
| **University of Stellenbosch Human Research Ethics Committee** | 00001372 | IRB0005240 |
| **South African Medical Research Council** | 00002753 | IRB00001569 |
